# Genome-wide identification of *GDPD* gene family in foxtail millet (*Setaria italica* L.) and functional characterization of *SiGDPD14* under low phosphorus stress

**DOI:** 10.3389/fpls.2025.1586547

**Published:** 2025-06-18

**Authors:** Chaomin Meng, Haojie Guo, Cheng Wang, Furong Yang, Bing Du

**Affiliations:** College of Agriculture, Henan University of Science and Technology, Luoyang, China

**Keywords:** foxtail millet, GDPD, gene family, low phosphorus stress, over-expression, functional characterization

## Abstract

Glycerophosphodiester phosphodiesterase (GDPD) catalyzes the hydrolysis of glycerophosphodiesters into sn-glycerol-3-phosphate (G-3-P) and corresponding alcohols, which is integral to various physiological processes in plants. However, our comprehension of the *GDPD* gene family in foxtail millet (*Setaria italica* L.) remains limited and unclear. This study aimed to identify and predict the function of *GDPD* gene family members in foxtail millet through a comprehensive genome-wide analysis.14 *SiGDPD* genes were identified in the foxtail millet genome. Phylogenetic analysis categorized *SiGDPD* proteins into five groups. Promoter regions of *SiGDPD* genes contained multiple *cis*-acting elements related to light response, hormone regulation, and stress response. Phylogenetic and collinearity analyses demonstrated conservation of GDPD proteins among foxtail millet, sorghum, rice, and maize, with the *SiGDPD* gene family undergoing purifying selection during evolution.Tissue differential expression analysis revealed distinct expression patterns of *SiGDPD* genes across various tissues, showing spatiotemporal expression characteristics. Under low phosphorus stress, the expression levels of *SiGDPD3* and *SiGDPD14* significantly increased, while *SiGDPD1*, *SiGDPD5*, *SiGDPD6*, and *SiGDPD11* showed significant decreases.To identify the function of *SiGDPD14*, an over-expressed transgenic *Arabidopsis* was generated. The results showed that transgenic *Arabidopsis thaliana* plants over-expressing *SiGDPD14* exhibited enhanced tolerance to low phosphorus stress.Taken together, the results of this study provide valuable information for further studies on candidate *SiGDPD* genes involved in the phosphate deficiency response in foxtail millet.

## 1 Introduction

Glycerophosphodiester phosphodiesterases (GDPDs), also referred to as GPX-PDEs, predominantly facilitate the catabolism of glycerophosphodiesters into sn-glycerol-3-phosphate (G-3-P) and their corresponding alcohol derivatives. These enzymes play a pivotal role in sustaining phosphate (Pi) homeostasis, particularly within phosphorus (P)-deficient environments (Ohshima et al., 2008; Cheng Y et al., 2011; Vander Rest et al., 2002). GDPDs specifically target glycerophosphodiesters—products arising from the deacylation of membrane phospholipids—including glycerophosphocholine (GPC), glycerophosphoethanolamine (GPE), glycerophosphoinositol (GPI), glycerophosphoserine (GPS), and glycerophosphoglycerol (GPG), contingent upon the nature of their head groups. In prokaryotes, they are produced by phospholipase A or B (Farn et al., 2001; Sitkiewicz et al., 2007), whereas in plants, glycerophosphodiesters are produced by lipid acyl hydrolases (LAH) from the deacylation of different membrane phospholipids, which are then substrates for GDPDs (Matos and Pham-Thi, 2009).

The initial identification of *GDPD* activity within plants was documented in the vacuoles and cell walls of carrot (*Daucus carota*), sycamore (*Acer pseudoplatanus*), and *Arabidopsis thaliana* cell suspension cultures. Following this discovery, *GDPDs* have been classified as a multigene family in Arabidopsis, delineated into two distinct groups: type A featuring a single GDPD domain (*AtGDPD1-6*) and type B characterized by two putative GDPD domains (*AtGDPDL1-7*). Type A *GDPDs* possess conserved catalytic residues akin to those found in *E. coli GlpQ*; conversely, type B *GDPDs* exclusive to plants exhibit diminished phosphodiesterase activity. Two *GDPD* genes, *GPX-PDE1* and *GPX-PDE2*, were also found in white lupin (*Lupinus albus*), which are capable of hydrolyzing glycerophosphodiester and are markedly upregulated by Pi deficiency, suggesting their involvement in phosphorus acclimation (Cheng L et al., 2011). Silencing these *GDPDs* inhibited root hair development under phosphorus deprivation, confirming that they participate in membrane lipid remodeling and root development during phosphorus starvation in plants (Wang et al., 2018).

The *GDPD* gene family has been rigorously studied across diverse plant species, encompassing Arabidopsis, rice (*Oryza sativa* L.), corn (*Zea mays* L.), and sorghum (*Sorghum bicolor* L.) in past research endeavors. Nevertheless, a comprehensive analysis solely focusing on the *GDPD* genes in foxtail millet remains conspicuously absent in contemporary literature. In the present study, we conducted an exhaustive genome-wide analysis of the *GDPD* gene family in foxtail millet, identifying 14 distinct members. This investigation delved into their phylogenetic relationships, conserved motifs and domains, *cis*-regulatory elements, collinearity and syntenic relationships, as well as their expression profiles across diverse tissues and phosphorus gradients, aiming to elucidate their potential biological functions within foxtail millet. Additionally, the functional characterization of *SiGDPD14* was undertaken through its over-expression in *Arabidopsis*. The insights garnered from this research augment our understanding of both genome-wide and functional analyses of the *GDPD* gene family.

## 2 Materials and methods

### 2.1 Plant materials and different treatment

Seeds of foxtail millet cultivar “Jigu13” were used in the present study. The seeds were sterilized in 75% ethanol for 5 min, dried, and germinated on moist plates for 3 days. Then, the seedlings of similar vigor were transferred into trays containing phosphorus-rich nutrient solution (Hoagland solution+1.0 mmol/L KH_2_PO_4_). The pH of the nutrient solution was adjusted to 5.8, and the solution was replaced every three days. The seedlings were grown in a chamber with a photoperiod of 12 h light/12 h dark, a day temperature of 28°C, and a night temperature of 22°C. When the seedlings were at the three-leaf stage, low phosphorus treatment was applied (Hoagland solution containing 1 μmol/L KH_2_PO_4_ + 1.0 mmol/L KCl), and the phosphorus-sufficient group was used as the control. To analyze the mRNA expression levels of *SiGDPD* gene, root samples were collected 8 h after treatment.

### 2.2 Identification of GDPD gene family in foxtail millet

Genome data and annotation files of foxtail millet were downloaded from MDSi (http://foxtailmillet.biocloud.net/home), and those of Arabidopsis were downloaded from TAIR (https://www.arabidopsis.org/) (Li et al., 2023). The query sequences were inputted from the protein sequences of *AtGDPD* family. TBtools was used to perform BLAST comparison in the total protein sequence of foxtail millet to identify candidate genes that belong to *SiGDPD* family with E-value < 1e−5 (Altschul et al., 1997). The HMM file of conserved domain of *GDPD* (PF03009) was downloaded from the Pfam database (http://pfam.xfam.org/) (Finn et al., 2011; Oelkers et al., 2008). The Simple HMM Search in TBtools software was used to screen the total protein sequence of foxtail millet to obtain the potential *SiGDPDs* genes. The candidate genes obtained by the two methods were merged to obtain the candidate genes of *SiGDPD* family. The candidate SiGDPD proteins containing GDPD domains were verified by SMART program (http://smart.embl-heidelberg.de/) and NCBI-CDD web server (https://www.ncbi.nlm.nih.gov/cdd/). The protein sequences containing functional domains identified by the two tools were cross-referenced, and the sequences without complete conserved domains were deleted to obtain the *SiGDPD* family members. Physicochemical properties including molecular weight and isoelectric point were analyzed by TBtools, and subcellular localization were predicted by WOLF (https://wolfpsort.hgc.jp/) (Chen et al., 2020).

### 2.3 Gene structure, conserved motifs, conserved domains and three-dimensional modeling analysis of GDPD genes in foxtail millet

The gene structure of the *SiGDPD* family was analyzed using the exon and intron information of *GDPD* family genes extracted from the foxtail millet genome annotation GFF file. Conserved motifs were identified through the MEME online tool (http://meme-suite.org/tools/meme), with a prediction value set to 12 (Bailey et al., 2009). The functions of domains and motifs in *SiGDPDs* were evaluated using the Batch Web CD-search. Furthermore, the gene structure view function of TBtools was employed to visually represent the distribution of conserved motifs, domains, and gene structures in *SiGDPD*. Additionally, tertiary structure prediction was conducted through homology modeling via SWISS-MODEL, available at http://swissmodel.expasy.org/interactive.

### 2.4 Phylogenetic tree construction, chromosomal localization and syntenic analysis of *SiGDPD*s

The full-length amino acid sequences of *GDPD* genes identified from *A. thaliana*, *O. sativa*, *Z. mays*, *S. bicolor* and *S. italica* were aligned using MEGA11 with default parameters. Subsequently, a phylogenetic tree was generated via the neighbor-joining method in MEGA11 with a bootstrap value of 1000 and default parameters (Rozewicki et al., 2019; Kumar et al., 2016). This evolutionary tree was further refined using the Evolview online tool, accessible at https://evolgenius.info/evolview/. The GTF/GFF3 format genome annotation file of *S. italica* was downloaded from the Phytozome database. TBtools software was used to depict the chromosomal localization of each *SiGDPD* gene. Interspecific and intraspecific collinearity analyses were conducted using GDPD protein sequences from *Arabidopsis*, rice, sorghum, maize, and foxtail millet. MCScanX software was used to analyze the gene duplication events in foxtail millet, and the results were visualized using TBtools.

### 2.5 Prediction of promoter *cis*−acting elements, protein-protein interaction and tissue expression pattern assay

The 2-kb upstream sequence of the foxtail millet *GDPD* gene was scrutinized using PlantCARE (http://bioinformatics.psb.ugent.be/webtools/plantcare/html) to identify *cis*-acting elements within the promoter region, including phosphorous responsive elements (e.g. PHR1 binding site),hormone regulatory, growth and development-related, and stress regulatory elements (Higo et al., 1999). The functional protein-protein interaction network model for GDPD proteins was developed utilizing the Web String database (https://string-db.org/), with a confidence parameter set to 0.400.Tissue-specific expression profiles of *SiGDPDs* were carefully investigated using the NCBI Short Read Archive database (https://www.ncbi.nlm.nih.gov/sra/) in different transcriptomic data cohorts. The heatmap analysis was performed with TBtools to generate gene expression heat maps on log2 scales.

### 2.6 qRT-PCR and expression analysis of *SiGDPD*s under low phosphorus stress

Total RNA was extracted from roots using trizol reagent (Invitrogen, USA) as directed by the manufacturer. First-strand cDNA was synthesized using Plant Reverse Transcription Kit (TIANGEN, China) and then diluted by tenfold to improve concentration accuracy. The corresponding primers were designed by Primer5 (**Supplementary Data Sheet 1**), and quantitative PCR was conducted on a fluorescent quantitative PCR instrument with three biological replicates and three technical replicates. The relative expression levels of RNA transcripts were calculated using the 2^−ΔΔCT^ method.

### 2.7 Generation of transgenic plants

The complete coding sequence of *SiGDPD14* was amplified, employing foxtail millet root cDNA as a template, and subsequently cloned into the pBI121-GFP plasmid. Transgenic *Arabidopsis* plants were then generated utilizing the inflorescence infection method. The regenerated plants underwent screening on a medium enriched with 50% hygromycin B and were cultivated under meticulously controlled conditions. Positive transgenic plants were ascertained through PCR, and their expression levels were quantified using qRT-PCR. Among the transgenic *Arabidopsis* lines, three overexpression lines of *SiGDPD14* (OE#2, OE#3, and OE#4) showing high expression levels were selected for detailed functional characterization. The primers employed for PCR and RT-PCR are delineated in **Supplementary Table S1**.

### 2.8 Analysis of transgenic plants resistance to low phosphorus stress

*Arabidopsis* seeds were sterilized using 75% ethanol for a duration of 5 minutes, followed by rinsing with distilled water. The seeds were transferred to 1/2 MS medium plates supplemented with either normal phosphorus (NP, 1 mmol·L^−1^ KH_2_PO_4_) or low phosphorus (LP, 1 μmol·L^−1^ KH_2_PO_4_), sealed with parafilm, and cultivated in a growth chamber under controlled conditions (22°C, 16/8 h light/dark cycle). After 14 days, the germination rate, root length, and root surface area of the *Arabidopsis* were measured.

### 2.9 Data processing and analysis

All experimental data were statistically analyzed and visualized using GraphPad Prism 8.0 (version 8.0; GraphPad Software, San Diego, CA, USA). Three independent biological replicates were performed for each experimental group. Statistical significance between datasets was evaluated by Student’s t-test, with asterisks indicating the following *p*-values:*: 0.01<p ≤ 0.05, **: p ≤ 0.01.

## 3 Results

### 3.1 Identification of *GDPD* genes in foxtail millet

In the foxtail millet genome, 14 *SiGDPD* genes were identified, designated as *SiGDPD1* through *SiGDPD14*. **Table 1** encompasses a detailed overview of these genes, presenting aspects such as gene ID, chromosomal location, genome position, amino acid length, and notable physicochemical characteristics. The amino acid sequences exhibited lengths ranging from 309 in *SiGDPD12* to an extensive 1097 in *SiGDPD13*. The isoelectric points (pI) spanned a spectrum from 5.02 for *SiGDPD6* to 8.88 for *SiGDPD12*, thereby classifying them into 3 alkaline and 11 acidic proteins. When examining molecular weights, values ranged between 34.14 kDa for *SiGDPD9* and 120.75 kDa for *SiGDPD13*. The analysis of GRAVY values revealed that 10 of the proteins possessed hydrophilic properties, in contrast to the remaining 4, which were hydrophobic. Further examination of subcellular localization indicated that 7 *SiGDPDs* were situated in the extracellular space, with the balance being found in the cytoplasm, plasma membrane, or nucleus. Signal peptide predictions highlighted the presence of signal peptides in 5 proteins within the *GDPD* family.

**Table 1:** Analysis of physicochemical properties of *GDPD* genes in foxtail millet.

Sequence ID	Gene name	Genomic location	Protein length (aa)	Molecular weight (KDa)	Isoelectric point(pl)	Signal peptide prediction	GRAVY^*^	Predicted subcellular localization
KQL28388 KQL28431 KQL29791	*SiGDPD1* *SiGDPD2* *SiGDPD3*	Chr1:5515835:5519327 Chr1:5854596:5863042 Chr1:24657945:24660823	362 1050 339	38.45 113.14 37.62	5.46 6.21 6.93	- + -	0.083 0.009 -0.131	Extracell Extracell Cytoplasm
KQL30247	*SiGDPD4*	Chr1:29336409:29342320	935	101.00	8.76	–	-0.058	Extracell
KQL26567	*SiGDPD5*	Chr2:45114734:45116030	355	40.23	5.55	–	-0.485	Plasma membrane
KQL11283	*SiGDPD6*	Chr4:32318970:32322315	622	66.44	5.02	–	0.085	Extracell
KQL07095	*SiGDPD7*	Chr5:37645570:37651977	552	62.02	6.17	–	-0.378	Plasma membrane
KQL02403	*SiGDPD8*	Chr6:32262585:32267866	768	82.94	5.93	+	0.008	Extracell
KQL02681 KQK97003 KQK97677 KQK94148 KQK95707 KQK88136	*SiGDPD9* *SiGDPD10* *SiGDPD11* *SiGDPD12* *SiGDPD13* *SiGDPD14*	Chr6:33940676:33943247 Chr7:18138918:18141808 Chr7:22594407:22599357 Chr8:9716155:9719516 Chr8:37373868:37381468 Chr9:11816810:11820073	312 395 747 309 1097 391	34.14 42.90 82.15 34.58 120.75 45.29	7.18 5.66 5.84 8.88 6.05 6.08	- - + - + +	-0.139 -0.130 -0.039 -0.221 -0.076 -0.432	Nucleus Nucleus Extracell Nucleus Cytoplasm Extracell

^*^The Grand Average of Hydropathy (GRAVY) is a calculated metric representing the sum of hydropathy values of all amino acids, divided by the number of residues present. In this context, positive values are indicative of hydrophobic tendencies, whereas negative values suggest the presence of hydrophilic properties.

### 3.2 Gene structure, conserved motif, conserved domain, and three-dimensional modeling analysis of *GDPD* genes in foxtail millet

**Figure 1:**
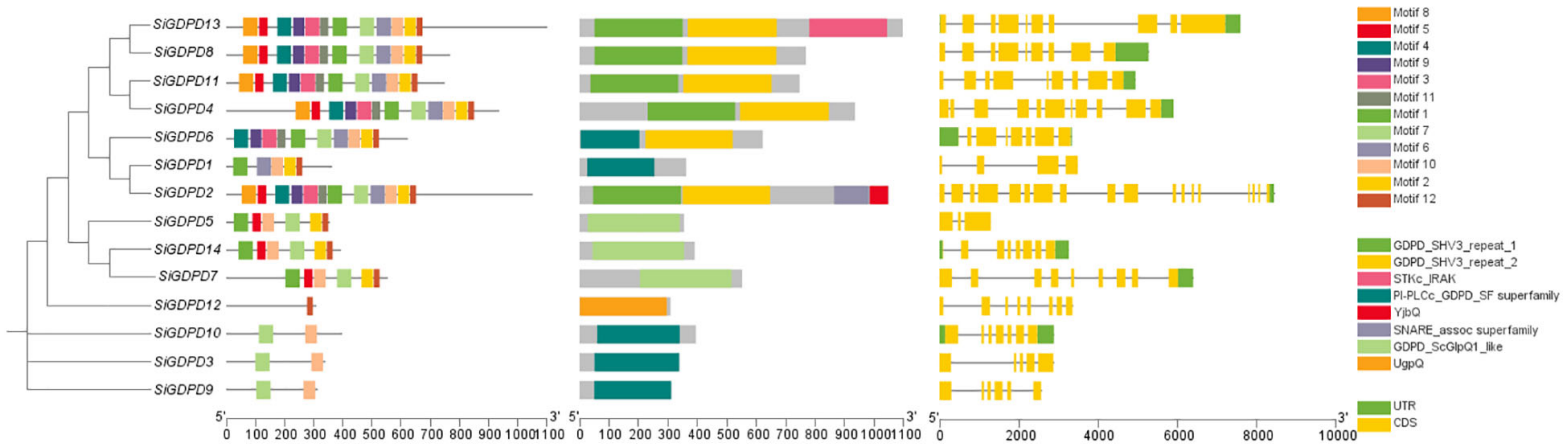
Conserved motifs, functional domain, and gene structure of 14 *SiGDPD* members in foxtail millet. These sizes could be estimated using the scale at bottom. **(A)** Gene tree. **(B)** Motif patterns.Conserved motifs in the *SiGDPD* peptides are presented by different colored boxes. **(C)** Conserved domain. GDPD domain is represented by box. **(D)** Gene structure. Coding sequences (CDS) and untranslated region (UTR) are represented by different colored boxes, and introns are represented by lines.

Using GFF annotation file of foxtail millet genome and CDS information of *SiGDPD* gene family, conserved domains and motifs and gene architectures of 14 *SiGDPDs* were studied using TBtools software based on the phylogenetic tree of SiGDPD protein sequences (**Figure 1**). Twelve conserved motifs were discovered in SiGDPDs using MEME online tool. Motif 1 and 6 are located in N-terminal GDPD domain while motif 12 is present in C-terminal GDPD domain. Further, structural characteristics of *SiGDPD* gene family were studied to understand their potential role. Exon–intron structure of SiGDPD genes is shown in the right panel of **Figure 1**. *SiGDPD2* has the highest number of introns (17) while *SiGDPD5* has the least number of introns (2).

**Figure 2:**
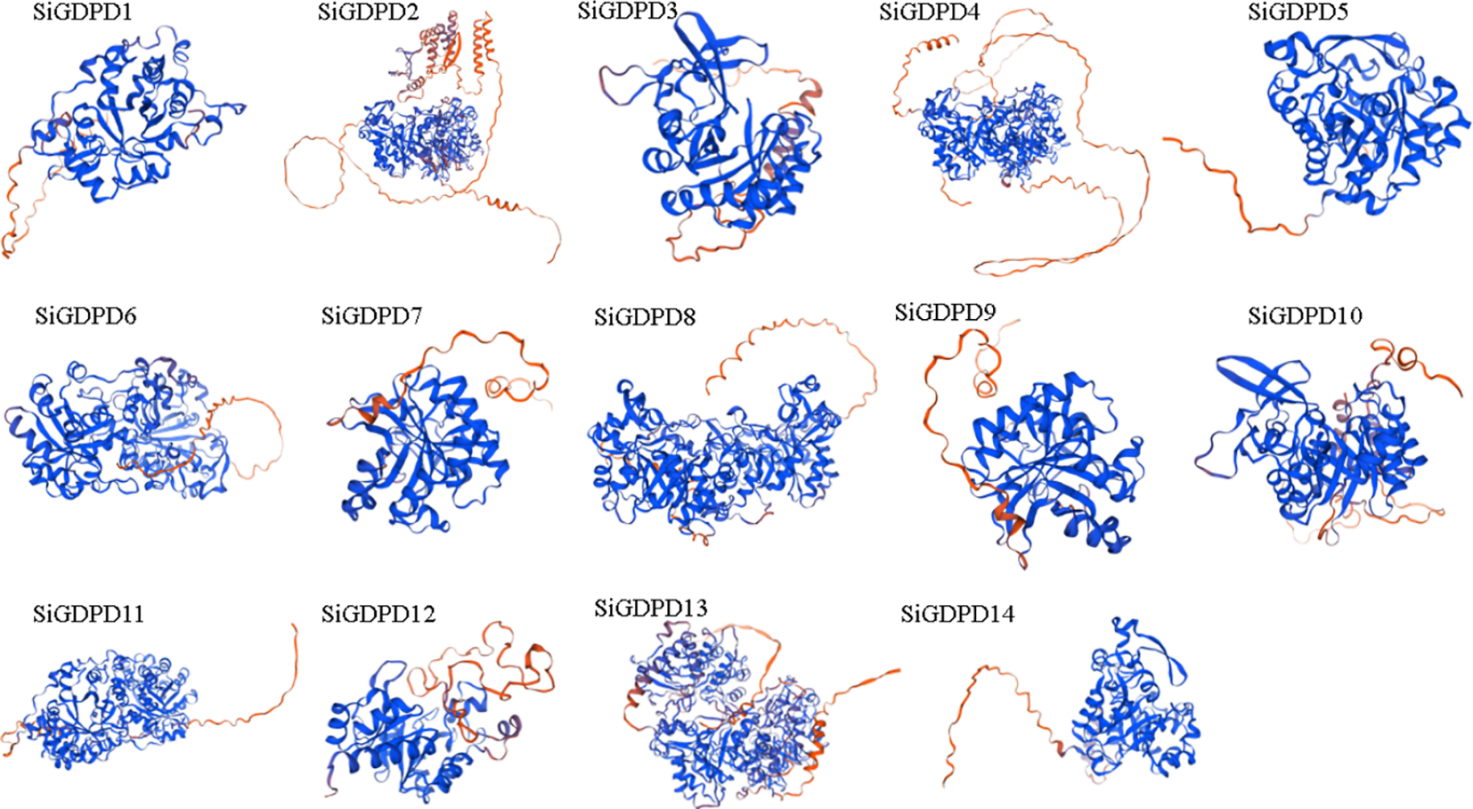
The three-dimensional models of GDPD proteins in foxtail millet. The red coloration indicates the α-helix or active site, while the blue coloration represents the β-sheet or positive charge region within the protein’s tertiary structure.

The three-dimensional structure modeling of SiGDPD proteins exposed the complexity of their folded structures, featuring alpha-helices, extended strands, beta-turns, and random coils (**Figure 2**). Despite the similar spatial configurations and conserved functional regions of all SiGDPD proteins, significant differences were noted in the sequences of N-terminal peptides. Those proteins closely related on the phylogenetic tree had similar motif compositions and gene structures (**Figure 1**), suggesting that they may perform similar functions in plant physiological processes as members of the same group.

### 3.3 Phylogenetic tree analysis of the *SiGDPDs*

**Figure 3:**
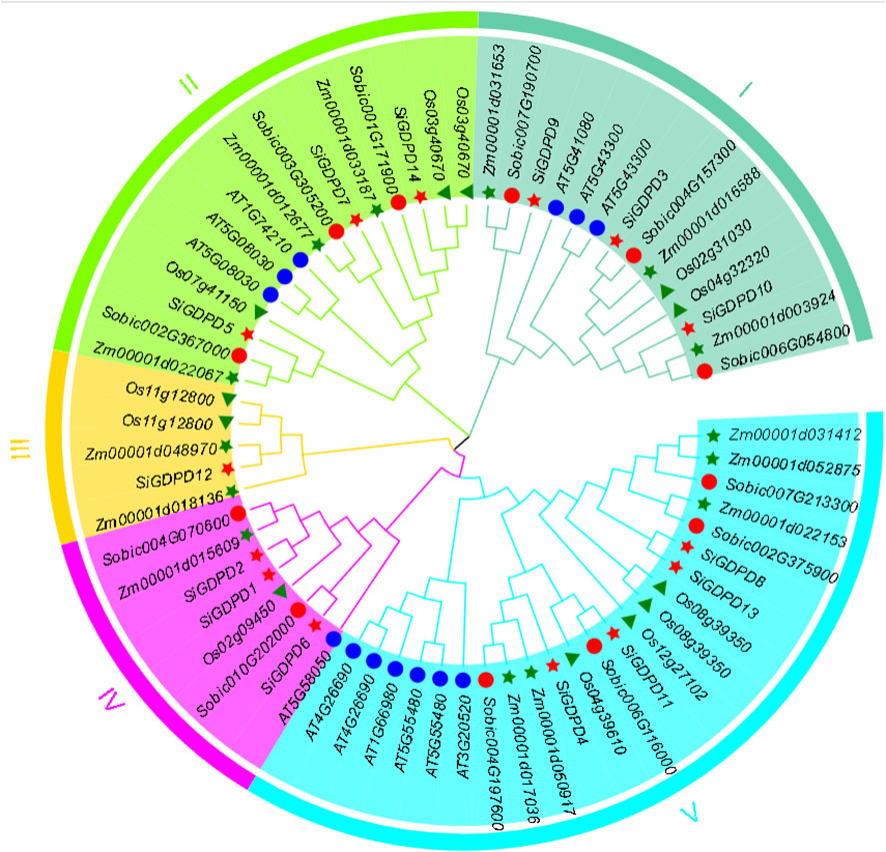
Phylogenetic relationships of GDPDs in *Setaria italica* (Si), *Arabidopsis*(AT), *Oryza sativa* (Os), *Zea mays* (Zm),and *Sorghum bicolor* (Sb).The phylogenetic tree was generated using the neighbor-joining method with MEGA 11 software. GDPDs in various species were marked with different labels and diverse subgroups of GDPDs were highlighted by different colors.

The evolutionary relationships of *SiGDPD*s in foxtail millet were explored using the full-length protein sequences of 13 GDPDs in *Arabidopsis*, 12 in rice, 14 in maize, 12 in sorghum, and 14 in foxtail millet, aligned with MEGA11. **Figure 3** illustrates the classification of these 65 GDPD proteins into five subgroups: Group I (14 genes), Group II (15 genes), Group III (5 genes), Group IV (8 genes), and Group V (23 genes). It is noteworthy that Group III does not include members from *Arabidopsis* or sorghum, while Group IV does not contain any members from *Arabidopsis*. Generally, the *GDPD* genes of foxtail millet demonstrate a closer evolutionary affinity to rice homologs than to those of *Arabidopsis*. Moreover, *GDPD* genes with similar genetic structures tend to cluster together.

### 3.4 Chromosome localization, gene duplication and syntenic analysis of *SiGDPDs*

**Figure 4:**
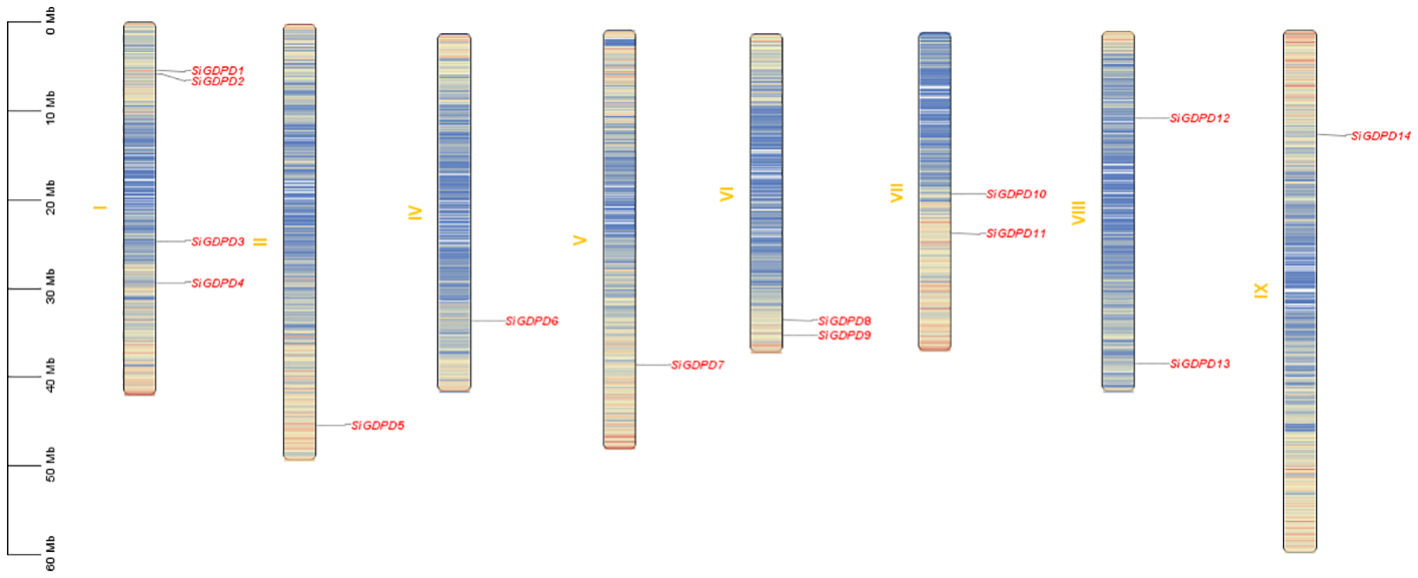
Distribution of 14 *SiGDPD* genes onto nine Setaria italica chromosomes. Graphical representation of physical locations for each *SiGDPD* gene on Setaria italica chromosomes. Chromosomal distances are given in Mb.

**Figure 5:**
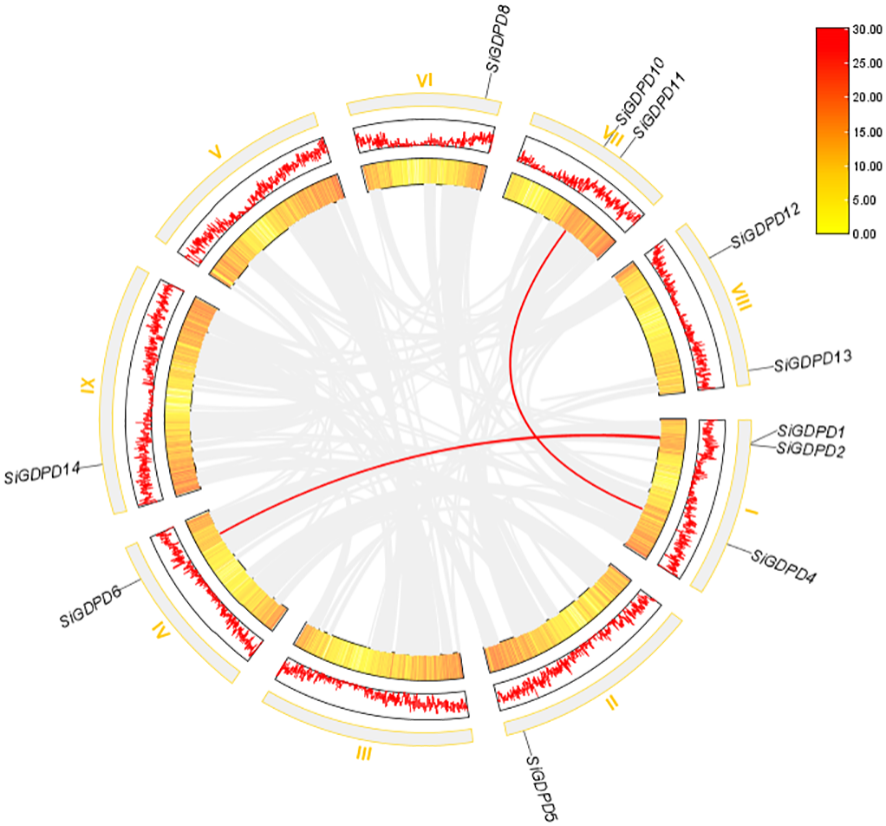
Gene duplication examination of *SiGDPD* genes.

**Figure 6:**
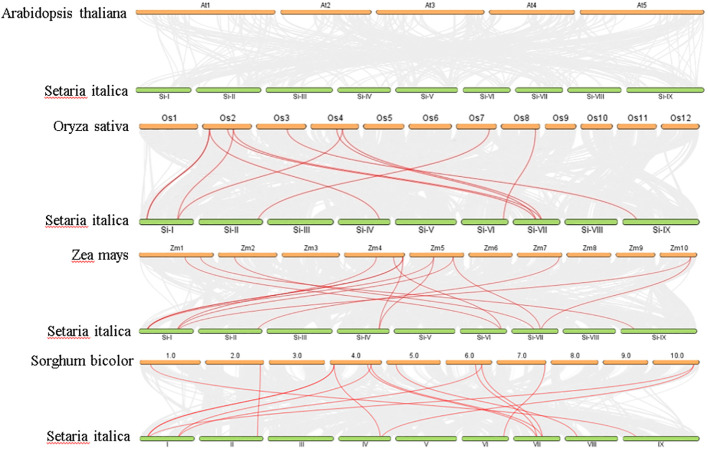
Synteny analysis of *GDPD* genes between *Setaria italica*, *Arabidopsis*, *Sorghum bicolor*, *Oryza sativa*, and *Zea mays*. Gray lines represent the collinear relationship between foxtail millet and four other species and red lines represent collinear *GDPD* gene pairs.

Analysis of chromosomal location revealed a non-uniform distribution of 14 *SiGDPDs* across eight of the nine chromosomes. Chromosome 1 had the highest number of *SiGDPDs*, comprising four members, while chromosomes 6, 7, and 8 each contained two genes. Chromosomes 2, 4, 5, and 9 were home to a single gene each, and chromosome 3 had none (**Figure 4**). Subsequent investigation focused on the duplication events of *SiGDPDs* in foxtail millet, identifying two duplicated gene pairs (*SiGDPD1*/*SiGDPD6* and *SiGDPD4*/*SiGDPD10*) (**Figure 5**), suggesting ancient tetraploidy events or whole-genome duplication (segmental duplication) as plausible causes. To elucidate the phylogenetic mechanism of *GDPD* genes across species, a homology analysis was performed comparing foxtail millet with four representative species: the dicotyledonous plant- *Arabidopsis*, and three monocotyledonous plants - *Oryza sativa*, *Zea mays*, and *Sorghum bicolor* (**Figure 6**). The highest homology was found in *Zea mays* and *Sorghum bicolor*, with 14 gene pairs, followed by *Oryza sativa* with 11 pairs, and none in *Arabidopsis*. The lack of homologous genes between foxtail millet and *Arabidopsis* implies their divergence post the split of monocotyledonous and dicotyledonous plants. Notably, the *GDPD* genes in foxtail millet exhibited the greatest homology with those in *Zea mays* and *Sorghum bicolor*, suggesting a common ancestral origin for these highly homologous genes among different plants.

### 3.5 Analysis of *cis*-acting elements in *SiGDPD* promoters

**Figure 7:**
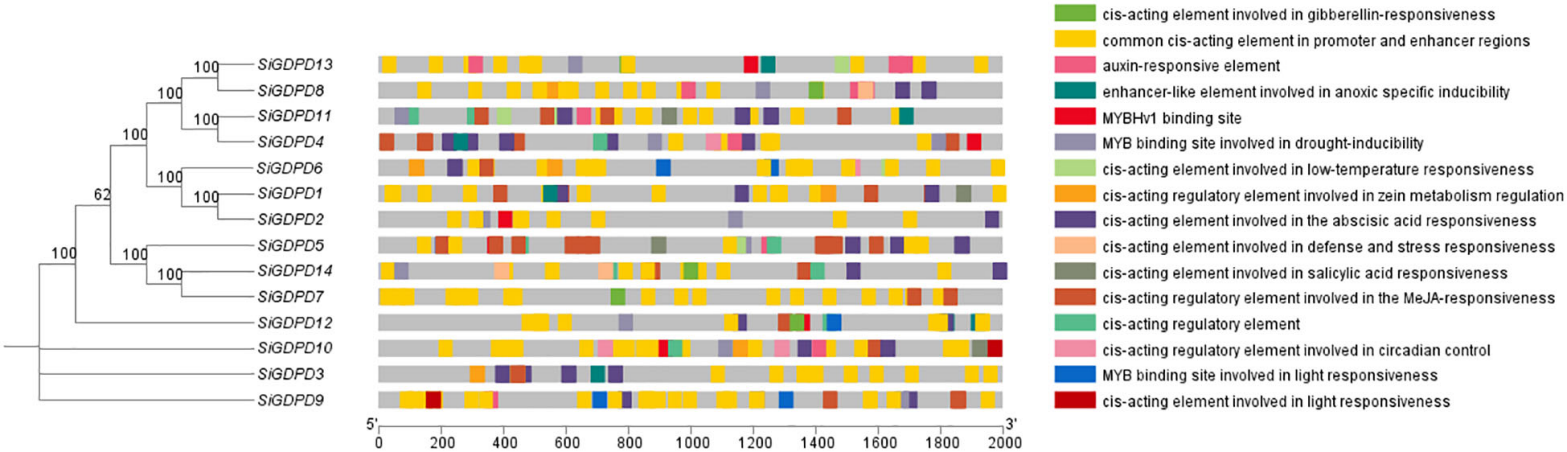
The *cis*-acting regulatory elements of the promoter region in the *GDPD* gene family. The different color blocks represent different types of *cis*-acting regulatory elements, and the gray lines refer to the upstream coding sequences.

To validate the regulatory roles of *SiGDPDs*, we analyzed the *cis*-acting elements in the promoter region (2000 bp upstream of the start codon of the *SiGDPDs*) (**Figure 7**). The *cis*-acting elements were categorized as light-responsive elements, hormone-responsive elements (auxin-, gibberellin-, abscisic acid- and salicylic acid-responsive elements), stress-responsive elements (low-temperature-, defense and stress- and drought-inducible elements) and development and growth-related elements. Although the different *SiGDPD* members had various *cis*-acting elements, there were photoresponsive elements in the promoters of all the *SiGDPDs*, suggesting that the *SiGDPDs* were light-inducible. Three *SiGDPDs* had low-temperature responsive elements, two had defense and stress responsive elements, and ten had drought responsive elements in their promoters, indicating that they might be involved in abiotic stress responses. A total of 14 *SiGDPDs* had at least one hormone response element in their promoters, which suggests that they might be regulated and expressed by multiple hormones. These results suggest that *SiGDPDs* are involved in many biological processes and play important roles in growth and stress responses in foxtail millet.

### 3.6 Prediction and correlation analysis of interacting proteins

**Figure 8:**
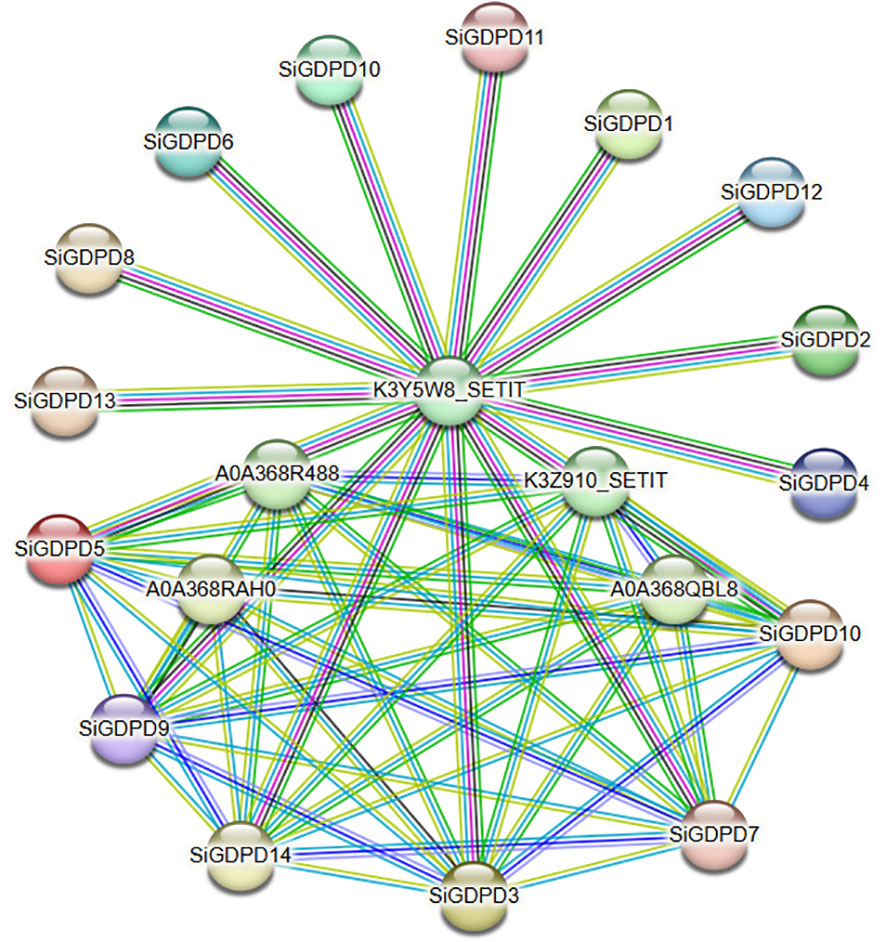
Prediction network of protein interactions for *SiGDPDs*. Each node represents a protein, and each edge represents an interaction.

Protein interaction networks provide significant insights for the prediction of functional orthologs within sequence homology clusters, thereby contributing to our understanding of gene interactions and regulatory relationships. In this study, we utilized the STRING online database to scrutinize interactions among individual *SiGDPDs*. The resulting network analysis revealed the co-expression of 14 *GDPD* family members with six associated proteins (**Figure 8**). Of particular interest was the observation that K3Y5W8_SETIT interacted with all 14 *SiGDPDs*, suggesting its potential role as a central protein within the family. Furthermore, *SiGDPD3*, *SiGDPD5*, *SiGDPD7*, *SiGDPD9*, and *SiGDPD10* were found to interact with each other, indicative of a potential functional correlation amongst these family proteins. These results offer valuable insights that can inspire further examinations aimed at eluci dating the roles of *SiGDPD* genes in diverse biological processes.

### 3.7 Gene expression analysis of *SiGDPDs* in different organs

**Figure 9:**
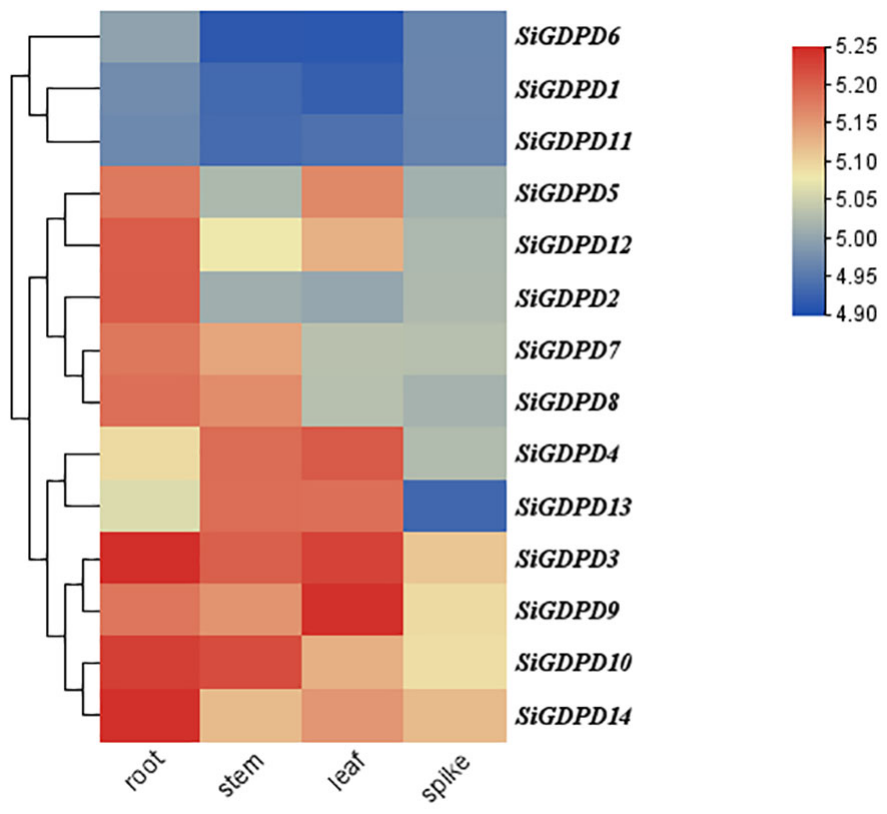
Heat map of *SiGDPD* genes expression patterns in different tissues. The red and blue blocks represent higher and lower expression levels of the genes, respectively.

Transcriptome data were employed to scrutinize the expression patterns of *SiGDPD* genes in various organs. *SiGDPD3*, *SiGDPD9*, *SiGDPD10*, and *SiGDPD14* consistently demonstrated high expression levels in all examined organs. In contrast, *SiGDPD1*, *SiGDPD6*, and *SiGDPD11* exhibited uniformly low expression levels. Organ-specific expression patterns were observed for certain genes: *SiGDPD2* and *SiGDPD12* were predominantly expressed in roots, *SiGDPD5* and *SiGDPD4* in leaves, and *SiGDPD4* and *SiGDPD13* in stems. Meanwhile, *SiGDPD7* and *SiGDPD8* displayed moderate expression levels across all organs and tissues (**Figure 9**).

### 3.8 Expression patterns of *SiGDPD* genes in response to low phosphorus stresses

**Figure 10:**
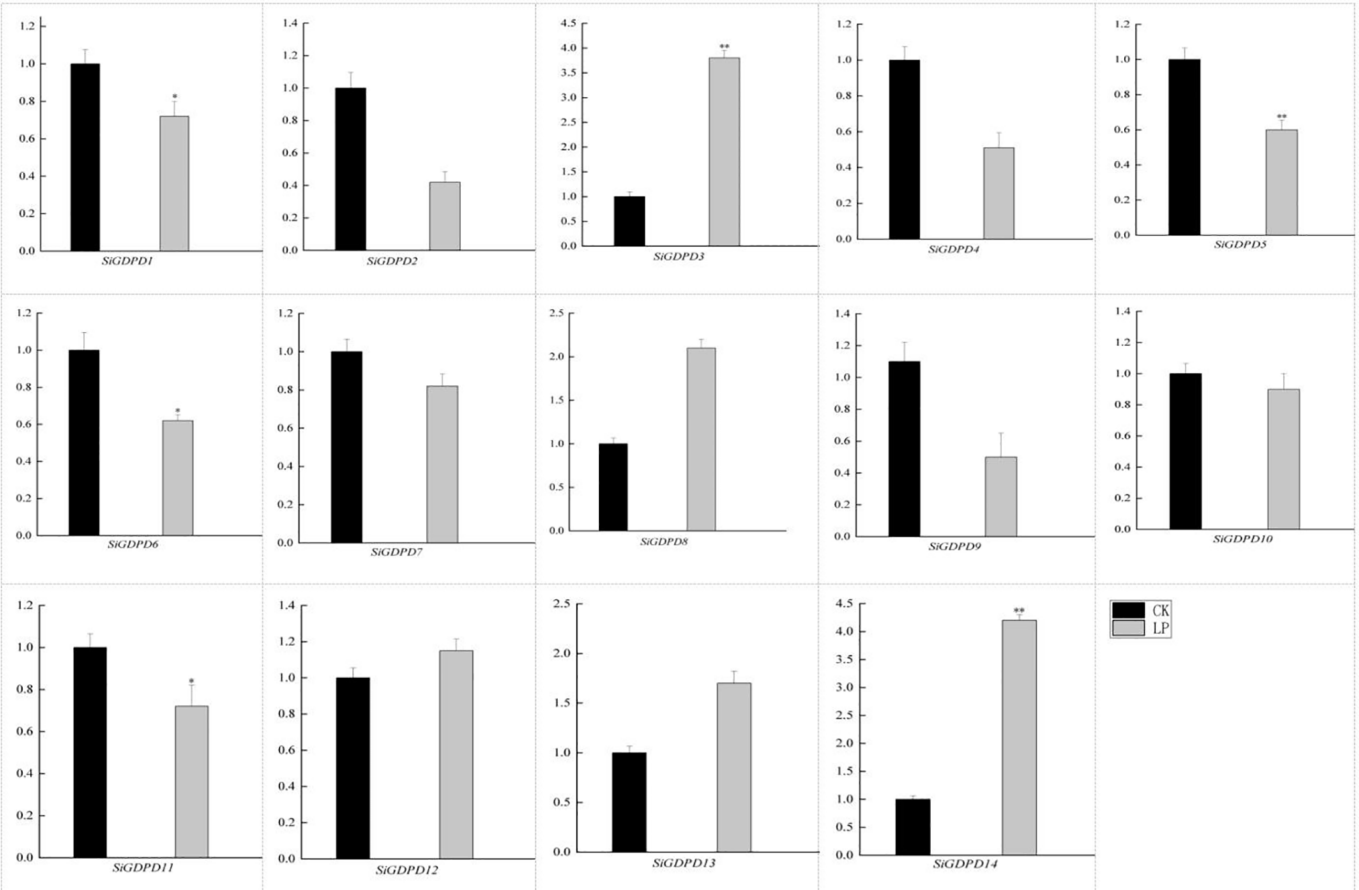
Gene expression analysis of the *SiGDPD* genes under low-phosphorus treatment. Black indicates the control group (CK) while Gray represents the low-phosphorus treatment group (LP). * and ** respectively indicate significant different (P < 0.05) and extremely significant different (P < 0.01) by student’s t test.

The *GDPD* gene family’s expression profiles were scrutinized in response to low phosphorus stress, utilizing qRT-PCR to analyze the expression levels of 14 *GDPD* genes under phosphate-deficient conditions. All 14 *GDPD* genes demonstrated altered expression levels compared to the control. Specifically, mRNA levels of *SiGDPD3* and *SiGDPD14* escalated significantly by 2.70-fold and 2.90-fold respectively under low phosphorus stress. Conversely, mRNA levels of *SiGDPD1*, *SiGDPD5*, *SiGDPD6*, and *SiGDPD11* decreased markedly by factors of 0.40-fold, 0.42-fold, 0.43-fold, and 0.41-fold respectively under phosphate-deficient conditions (p<0.05; **Figure 10**). These results suggest that *SiGDPD3*, *SiGDPD14*, *SiGDPD1*, *SiGDPD5*, *SiGDPD6*, and *SiGDPD11* may play a pivotal role in regulating the response to phosphate deficiency in foxtail millet.

### 3.9 Response of transgenic *Arabidopsis thaliana* over-expressing to low phosphorus stress

**Figure 11:**
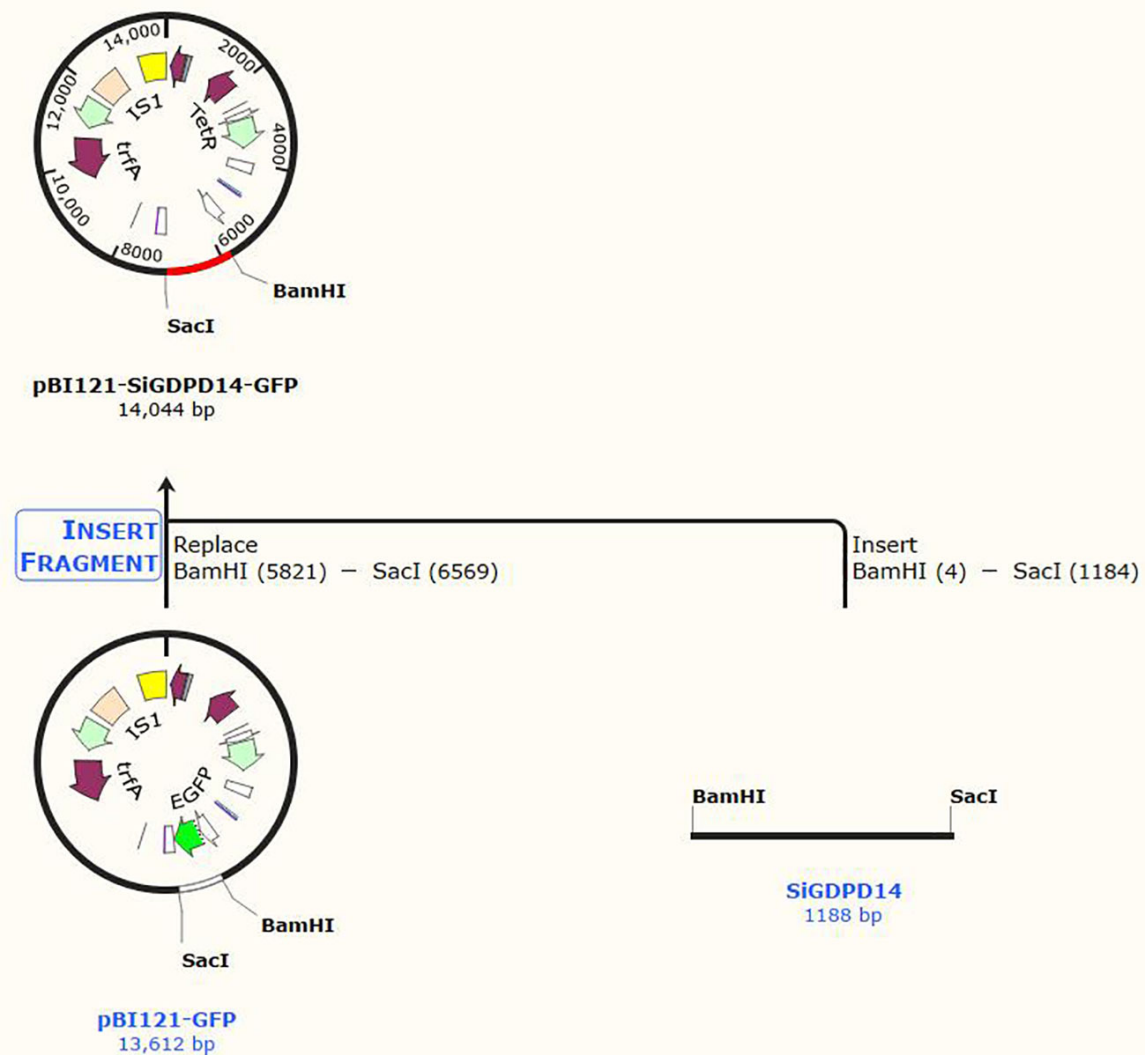
Genetic transformation of *SiGDPD14* in *Arabidopsis*. pBI121-*SiGDPD14*-GFP fusion protein construct.

**Figure 12:**
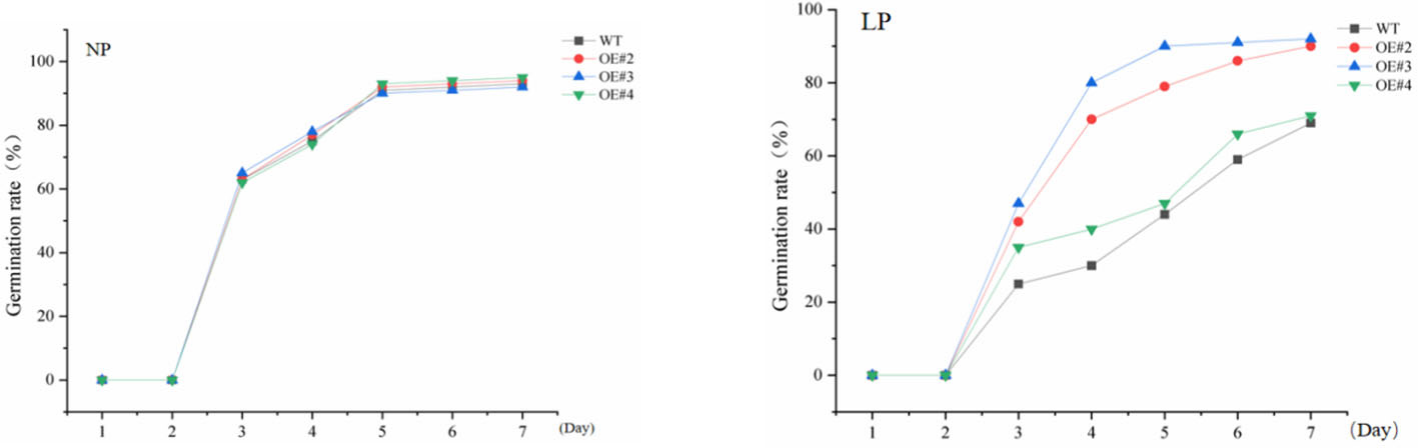
Germination rate of wild-type (WT) and *SiGDPD14* transgenic lines (OE#2, OE#3, and OE#4). Data were quantified using three biological replicates of each cultivar. Each data point represents the mean (± SD) of three separate experiments (p < 0.05).

**Figure 13:**
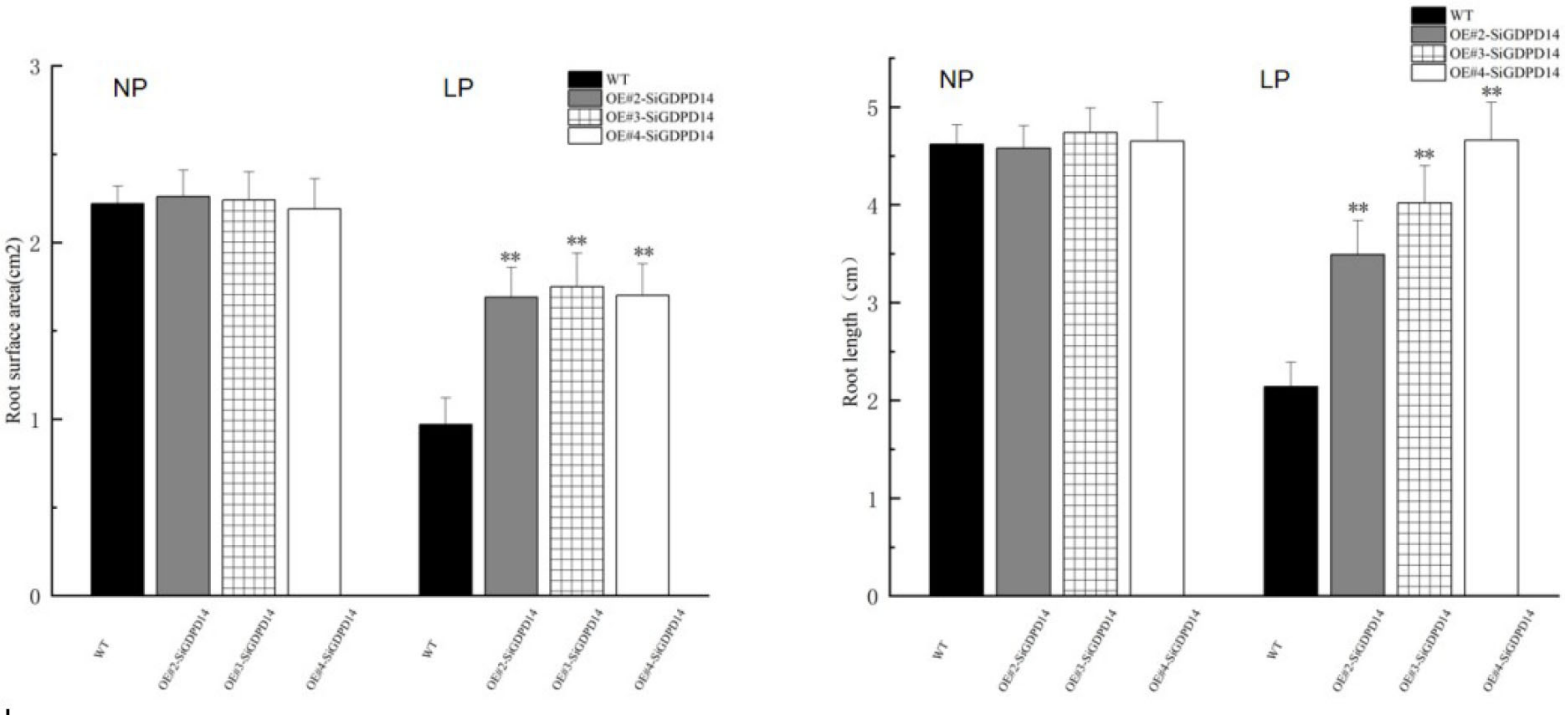
The primary root length and root surface area of WT and sigdpd14 transgenic line (OE#2, OE#3, OE#4) seedlings under NP and LP treatments were measured. All experiments included three replicates.Error bars represent standard deviations. Significant differences are indicated: ‘**’,p < 0.01 (Student’s t-test).

To explore the significance of *GDPD* genes during stress response, *SiGDPD14* was ectopic expression in *Arabidopsis* and three transgenic lines (OE#2, OE#3 and OE#4) were used to detect the response to low phosphorus stress (**Supplementary Data S5**, **Figure 11**). Under normal conditions, there was no discernible difference in seed germination between the wild-type and transgenic plants during the germination stage (**Figure 12**). However, under low phosphorus (LP) treatment, the seeds of the transgenic lines exhibited a significantly higher germination rate compared to the wild type. Specifically, only 30% of the wild-type seeds germinated, while the germination rates for the OE#2, OE#3, and OE#4 transgenic lines were 70%, 80%, and 40% respectively. Under normal conditions, there was no significant difference in root length and root surface area between the transgenic seedlings and the wild type (**Figure 13**). However, after 14 days of LP treatment, the average root length of the transgenic lines was 4.6 cm, which is 2.12 times longer than that of the wild-type plants. Similarly, the root surface area was 1.75 times larger than that of the wild-type. These results demonstrate the enhanced low phosphorus tolerance of the *SiGDPD14* transgenic lines.

## 4 Discussion

Glycerophosphodiester phosphodiesterases (GDPDs), also known as GPX-PDEs, primarily catalyze the conversion of glycerophosphodiesters into sn-glycerol-3-phosphate (G-3-P) and their corresponding alcohols. These enzymes contribute significantly to maintaining phosphate (Pi) homeostasis, particularly in phosphorus (P)-deficient conditions (Ohshima et al., 2008; Cheng Y et al., 2011; Vander Rest et al., 2002; Cheng L et al., 2011). Despite this, there are no existing reports discussing the role of the *SiGDPD* gene in the emerging C4 model crop, foxtail millet. To address this gap, a systematic study of the *SiGDPD* gene is imperative. In this study, we identified 14 *SiGDPD* members and analyzed their gene structure, conserved motifs, phylogenetic evolution, chromosome localization, collinearity, *cis*-elements, and gene expression patterns. Simultaneously, the root-specific expression gene *SiGDPD14* was heterologously transformed into the dicotyledonous model plant *Arabidopsis thaliana*. This provides insights into the role of the *GDPD* gene in plant growth and development, which can be beneficial in understanding its function in foxtail millet.

### 4.1 Identification and analysis of *SiGDPD* genes in foxtail millet

A total of 14 *SiGDPD* members were identified in this study. The physicochemical properties of 14 *SiGDPD* genes were analyzed, revealing that the relative molecular weight ranged from 34.14 to 120.75 kDa and the isoelectric point varied between 5.02 and 8.88. Based on the subcellular localization of the GDPD protein, the majority of SiGDPD proteins are present in the plasma membrane or extracellular space. Previous studies have suggested that GDPD transcription factors (TFs) are primarily localized to the nucleus, which is in contrast to our findings. This discrepancy may be attributed to potential transport mechanisms of TFs from the nucleus to the cytoplasm through nuclear transport signal domains within protein sequences, or post-translational modifications such as phosphorylation, acetylation, or methylation (Macfarlane et al., 1999). However, these hypotheses have not been confirmed for GDPD TFs. Furthermore, the bioinformatics-predicted subcellular localization of these TFs necessitates experimental validation. Analysis of conserved motifs (**Figure 1B**) revealed that all SiGDPD proteins, except SiGDPD12, contain motif 10, and the majority also possess motifs 7 and 12. This conservation indicates the functional significance of motifs 7, 10, and 12 across SiGDPD proteins. The arrangement of exon-intron structure contributes to the evolutionary dynamics of gene families, with introns enhancing protein diversity through exon shuffling and alternative splicing (Gorlova et al., 2014; Liu et al., 2019). Our analysis revealed that *SiGDPD* genes within the same group share similar exon-intron structures (**Figure 1D**), suggesting distinct biological functions for *SiGDPD* genes in different groups.

The gene promoter is the DNA sequence situated upstream of the gene coding region. It is composed of numerous *cis*-acting elements and functions as a specific binding site for proteins, which are accountable for initiating and regulating the transcription process (Hernandez-Garcia and Finer, 2014). The control of gene expression by *cis*-elements in the promoter region has emerged as the principal mechanism for organisms to adjust to diverse environments (Walther et al., 2007). In this study, we identified an array of *cis*-acting elements in 14 *SiGDPD* genes. The majority of these *SiGDPD* genes contain light response-related elements, hormones (including auxin, gibberellin, abscisic acid, and salicylic acid), stress response, and various other *cis*-acting elements related to growth and development within the *SiGDPD* promoter region. The phosphate starvation response is influenced by both internal and external factors, such as light and plant hormones. Prior research has highlighted the pivotal role of light as a signal to trigger the phosphate starvation response. Moreover, light is a key regulator of plant development and growth, impacting subterranean processes like phosphate uptake and assimilation (Liu et al., 2017; Sakuraba et al., 2018). Consequently, we hypothesize that the *cis*-acting elements associated with light response in the *GDPD* promoter could play a critical role in the adaptation mechanism of foxtail millet to phosphate starvation.

### 4.2 Evolution of *SiGDPD* genes

The majority of repetitive events in the foxtail millet genome are found in genome-wide replication events common to gramineous plants (Zhang et al., 2012). To investigate the evolutionary relationships within the *GDPD* gene family across different crops, we performed a collinearity analysis with foxtail millet, *Arabidopsis*, sorghum, maize, and rice. The analysis revealed that most *GDPD* genes exhibited syntenic relationships with genes in gramineous crops such as maize, sorghum, and rice. However, no collinearity was observed with *Arabidopsis* (**Figure 6**). Additionally, phylogenetic analysis showed a strong affinity between *SiGDPD* genes and their orthologs in *ZmGDPD* and *SbGDPD* genes (**Figure 3**). This indicates that the *GDPD* gene is highly conserved in gramineous plants and may experience various gene replication, loss, or functional differentiation events during the evolution of dicotyledonous and monocotyledonous plants (Lynch and Conery, 2000). To further elucidate the evolutionary constraints on the *SiGDPD* gene family, we analyzed non-synonymous and synonymous substitutions in S*iGDPD*. Our results show that the Ka/Ks ratio, a metric of selective pressure, was less than 1 for *SiGDPD* genes, indicating purifying selection. Taken together, these findings suggest the conservation and expansion of the *GDPD* gene family throughout evolution.

### 4.3 *GDPD* plays an important role in maintaining phosphorus homeostasis

A substantial body of evidence demonstrates the crucial role that GDPD TFs play in regulating phosphorus homeostasis in plants. Under conditions of phosphorus restriction, *AtGDPD1* participates in the hydrolysis of deacyl phospholipases, such as glycerophosphoglycerophosphate, with the resultant G-3-P potentially being dephosphorylated via a GPP-mediated pathway to release phosphorus (Cheng Y et al., 2011). *OsGDPD2* contributes to rice’s low-phosphorus tolerance by encouraging root growth and facilitating phosphorus release from cellular phosphorus pools (Poonam et al., 2019). Additionally, GDPD transcript levels have been observed to gradually accumulate during prolonged phosphorus deficiency in both rice and chickpea (Mehra et al., 2019; Zhang et al., 2007). Notably, most *ZmGPX-PDE* transcription levels are upregulated under phosphorus deficiency, a trend that is accompanied by an increase in *ZmGPX-PDE* activity (Wang et al., 2021). These findings suggest that phosphorus homeostasis is primarily maintained through the *GDPD* gene-mediated hydrolysis of phospholipids. Our research found that the over-expression of *SiGDPD14* in *Arabidopsis* led to longer roots and increased fresh weight, suggesting that the *GDPD* gene family in foxtail millet may function similarly to that in *Arabidopsis* in maintaining phosphorus homeostasis.

## 5 Conclusion

**Figure 14:**
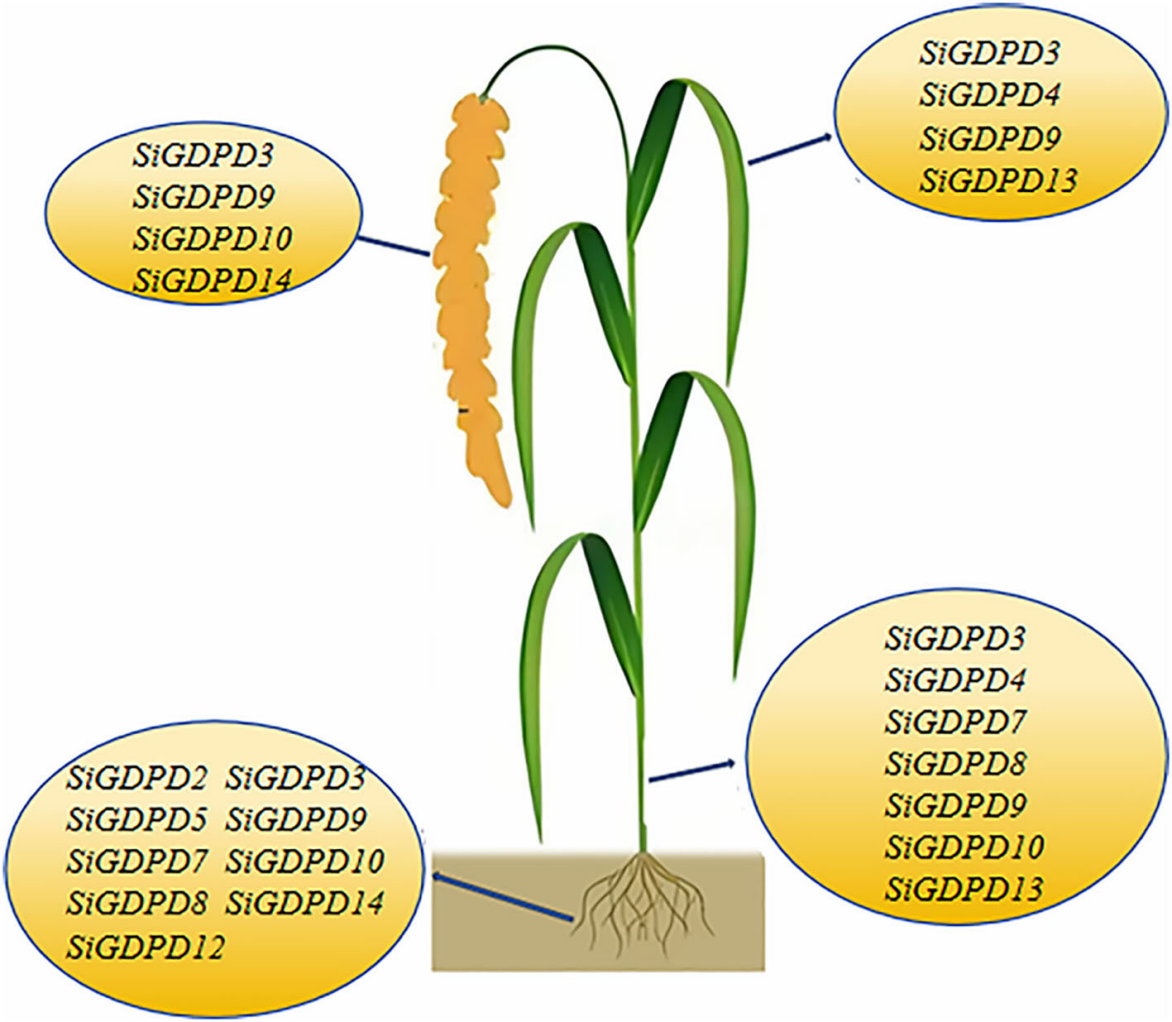
Model of the *SiGDPD* genes responding to low phosphorus stress in foxtail millet.

This study identified 14 *SiGDPDs* via genome-wide analysis, examining their phylogenetic relationships, gene and protein structures, conserved motifs, chromosomal locations, gene duplication events, promoter *cis*-acting elements, and expression patterns under low phosphorus stress conditions. The findings suggest that the *SiGDPD* family can be divided into five groups, with an uneven distribution of the 14 *SiGDPDs* across eight chromosomes. Gene duplication analysis indicated tandem duplication as the primary mechanism driving *SiGDPD* family expansion, while purifying selection appears to be the main influence on the family’s evolution. The prediction of *cis*-acting elements unveiled several stress-responsive elements. Analysis of tissue specificity revealed different genes exhibiting varying levels of expression in different tissues (**Figure 14**). Results from qRT-PCR demonstrated significant changes in the expression levels of all 14 *SiGDPD* genes in root tissue following phosphorus starvation, particularly for *SiGDPD3*, *SiGDPD1*, *SiGDPD5*, *SiGDPD6*, *SiGDPD11*, and *SiGDPD14*. Furthermore, the heterologous expression of SiGDPD14 enhanced the tolerance to low phosphorus stress in transgenic *Arabidopsis*. These findings suggest that *SiGDPD* genes perform diverse biological functions and may regulate foxtail millet’s tolerance to low phosphorus stress. This comprehensive analysis of the *SiGDPD* gene family provides valuable insights for further understanding of their biological roles and identification of potential candidate genes for low-phosphorus tolerant breeding programs.

## Data Availability

The datasets presented in this study can be found in online repositories. The names of the repository/repositories and accession number(s) can be found in the article/**Supplementary Material**.
