# Supplementary material for: Genome-wide identification of GDPD gene family in foxtail millet (Setaria italica L.) and functional characterization of SiGDPD14 under low phosphorus stress
Source: Front Plant Sci. 2025 Jun 18;16:1586547. doi: 10.3389/fpls.2025.1586547 (PMC12213840; doi:10.3389/fpls.2025.1586547)
Supplement: Supplementary file 1 [file DataSheet1.pdf]

Arabidopsis thaliana

>AT3G02040

MSLKAIHVSEVPSLDHFPENPSLICSSRKANNKFVVVGHRGHGMNMSQSPDLRFSALKENSIL
SFNAASKFPLDFIEFDVQVTRDGCPIIFHDDFIYSEEQGVVYEKRVTEVCLSEFMSYGPQRDTG
KTGKPLLRKSKEGKIHKWSVATDDSFCTLQEAFEKVENPNLGFNIELKLDDNVFYSSDHLSRL
LLPILQVVSDIGNDRTIIFSSFHPDAALLVRKLQTTYPVFFLTNGGTEMYHDTRRNSLEEAIKV
CLEGGLQGIVSEVKGVFRNPALVNKIKESKLSLMTYGKLNNVAEAVYMQHLMGIEGVIVDH
VEEITEAVREMMKPSNRDADGTKPKPNFSDRELSFLLKLIPELIQH*

>AT5G41080

MALRTVLVSDVPSLPDSVYGLSEGLELSKPTSFRLPGFSVIGHRGIGMNVLQSSDRRARGVKE
NSILSFNSAAKYPIDFIEFDVQVTKDDCPVIFHDDFIYSEENGIVNESRVTDLSLSEFLLYGPQKE
TEKIGKTLMRKSKEGKVLKWDVDLDDSLCTLQEAFEQVEQTLGFNIELKFDDQTVYEREFLV
HILRSVLQVVSNYAKDRPVIFSSFQPDAAKLVRELQSTYPVFFLTDAGNEIHNDERRNSLEEAI
QVCLEGGLQGIVSEVKGVFRNPAAISKIKESNLSLLTYGKLNNVGEAVYMQYVMGIDGVIVD
FVEEIIESTTRMMIRPPPSSSPLPSPSKDDDVAITRPEFSQKEISFLLKLLSQLIQH*

>AT5G43300

MALETMTLSLSSSAMLSSGVVEDDKKQEAIVFPKFVLMGHRGFGMNMLQSPDEKMKFIKEN
SLLSFNVAADFPIDFIEFDVQVTRDGCPVIFHDIFMFTQEQGVIIEKRVTEMDLHEFLSYGPQRD
GTNVKPMWRKTKDGRIFEWKVEKDDPLCTLEDAFLNVKHSLGFNIELKFDDNTVYGEGELR
QTLDNILTVVNEHSKNRPIIFSSFHPDAARLIRNMQRCYPVFFLTNGGCEIYKDVRRNSLDEAI
KLCKESGLQGLVSEVKAILRTPNAITRVKDSKLSLLSYGQLNNVVEVIYLQYLMGVEGVIVD
MVKDISEAIANIEVTNEDDCEGEDERKCLIRFGEERKKVEITKDMITLLNKFVPKLL*

>AT1G71340

MAIFEWRHRRRPFDGGGTRRRRFFSPLYSRNFKRTILFAVIFLAIFPPLYFHFKLRRIRQIVAQK
CDWLHHPPLVCAHGGDSTLAFPNTMDAYSFAIRSRVDCIEVDVSRSSDGVLFALHNRDLQRI
ARNSSVQVGDLSMKQIKELDVSEIVKGTLGSSRIPTLEEALALISNSVRKVILDAKVGPPMYEK
GLAQDILSIIERAQCNNCIVWAKSDTLARDIIRRAPDTMVGYIVMVDPLTGARNSLLRMKGAR
VVGVYHPLIDEELVRVVRRRNKEVYAWTVDDADPMKRMLHLGVDAVVTSDPSMFQGLME
DLRTECLEEGFSIRT*

>AT1G74210

MILTRCLPLIWLSLLTVCAAGRTLHPLPVKGPKTVKLQLQTSRPYNIAHRGSNGEIPEETTAAY
LKAIEEGTDFIETDILSSKDGVLICFHDCILDETTNVASHKEFADRKRTYDVQGFNITGFFTFDF
TLKELKQLRIKQRYAFRDQQYNGMYPIITFEEFLTIARDAPRVVGIYPEIKNPVLMNQHVKWP
GGKKFEDKVVETLKKYGYGGSYLSKKWLKKPLFIQSFAPTSLVYISNLTDSPKVLLIDDVTMP
TQDTNQTYAEITSDAYFEYIKQYVVGIGPWKDTIVPVNNNYVLAPTDLVKRAHAHNLQVHP
YTYRNEHEFLHYNFSQDPYKEYDYWINEIGVDGLFTDFTGSLHNFQEWTSPLPDTSKSPRQLL
SQIASLVLPYAKA*

>AT5G08030

MQCGRWRNFSSSNSVMAFKYLLPLLLLSLLVANCASRPLYRLPSEAKHATKKPLQTSRPYNL
AHRGSNGELPEETAPAYMRAIEEGADFIETDILSSKDGVLICHHDVNLDDTTDVADHKEFADR
KRTYEVQGMNMTGFFTVDFTLKELKTLGAKQRYPFRDQQYNGKFPIITFDEYISIALDAPRVV
GIYPEIKNPVFMNQQVKWADGKKFEDKFVETLKKYGYKGSYLSEDWLKQPIFIQSFAATSLV
YISNMTDSPKLFLIDDVTILTEDTNKTYAEITSDAYLDYIKPYVIGIGPWKDTIVPVNNNRLMT
PTDLVARAHSRNLQVHPYTYRNENQFLHLEFNQDPYLEYDYWLNKIGVDGLFTDFTGSLHN
YQELKSPLPQQQ*

>AT1G66970

MNSRPSNPTKLVIRSSTLLFCGVVLIHLFAAQIDAQRSTSRWQTLNGESCSHFISFFCALFPRKQ
ENLCDAPLVIARGGFSGLYPDSSIAAYQLATLTSVADVVLWCDLQLTKDGLGICFPDLNLAN
ASTIDRVYPNREKSYSVNGVTTKGWFPNDFSLTELQNFLLIRGILSRTDRFDGNGYLISTIEDV
VTTLNREGFWLNVQHDAFYEQQNLSMSSFLLSVSRTVSIDFISSPEVNFFKKITGSFGRNGPTF
VFQFLGKEDFEPTTNRTYGSILSNLTFVKTFASGILVPKSYILPLDDEQYLVPHTSLVQDAHKA
GLQVYVSGFANDVDIAYNYSSDPVSEYLSFVDNGDFSVDGVLSDFPITASAAVDCFSHIGRNA
TKQVDFLVISKDGASGDYPGCTDLAYEKAIKDGADVIDCSVQMSSDGVPFCLRSIDLRNSIAA
LQNTFSNRSTSVPEISSVPGIFTFSLTWPEIQSLTPAISNPFRVYRIFRNPREKNSGKLISLSQFLD
LAKTYTSLSGVLISVENAAYLREKQGLDVVQAVLDTLTEAGYSNGTTTTKVMIQSTNSSVLV
DFKKQSKYETVYKIEETIGNIRDSAIEDIKKFANAVVINKDSVFPNSDSFLTGQTNVVERLQKS
QLPVYVELFRNEFVSQAYDFFSDATVEINAYIYGAGINGTITEFPFTAARYKRNRCLGREEVPP
YMLPVNPGGLLNVMSPLSLPPAQAPNQDFIEADVTEPPLSPVIAKAPTSTPGTPSTIAQAPSGQ
TRLKLSLLLSVFFLSLLLL*

>AT1G66980

MNSQQSTRTKQMLQQSSTHLLCGVVLLQLFAAQVDAQRSTSPWQTLSGDAPLVIARGGFSG
LFPDSSLAAYQFAMVVSVADVVLWCDVQLTKDGHGICFPDLNLANASNSEEVYPNRQKSYP
VNGVTTKGWFPIDFSLTELQKVLFSLIRGILSRSGKFDENGYSISTVQNVATQMKPALFWLNV
QHDEFYEQHNLSMSSFLLSTSRTVSIDFISSPEVNFFRKIAGGFGNNGPSFVFQFMGKEDFEPTT
NRTYGSILSNLSFVKTFASGILVPKSYILPLDDKQYLLPHTSLVQDAHKAGLKLYASGFANDV
DIAYNYSWDPVSEYLSFVDNGNFSVDGMLSDFPLTASASVDCFSHIGRNATKQGNECNLLYIS
FLFLLIEQKHYHLPMVRASVDFLVISKNGASGEYPGCTKLAYEKAIKDGSDVIDCPVQMSSDG
IPFCSSSIDLVNSTTVGQTHLRNRSIIVPEISSVAGIFTFSLTWHEIQSLTPAISNPFRENGMSRNP
NERNSGNLISLYEFLNLAKNSTSLSGILISLENVVYLREKKGLDVVKVVLNRLTETGYIVGTLK
VMIQSTTRLVLVDFKNQSTYKTVYKIKETIGNITDSAIEDIKKFANAVVINKASVFPNSDSFLT
GQTTNVLERLQKFQLPVYVELFQNEFVSQPFDFFADETVEINAYIFGAGINGTITEFPYTAARY
KRNRCLGREEVPPYMLPVNPGGVLTLISTSSLPPAQDPNPIFTHDDVTEPPLPPVIAKSPTSTLG
TPSTIAKPLRNFLKVIRIVSWSVAGVVLFLVLLTLVFCFHRKRETRLRQQKLKALIPLEHYTYA
QVKRITKSFAEVVGRGGFGIVYKGTLSDGRVVAVKVLKDTKGNGEDFINEVATMSRTSHLNI
VSLLGFCSEGSKRAIIYEFLENGSLDKFILGKTSVNMDWTALYRIALGVAHGLEYLHHSCKTRI
VHFDIKPQNVLLDDSFCPKVSDFGLAKLCEKKESILSMLDTRGTIGYIAPEMISRVYGNVSHKS
DVYSYGMLVLEIIGARNKEKANQACASNTSSMYFPEWVYRDLESCKSGRHIEDGINSEEDEL
AKKMTLVGLWCIQPSPVDRPAMNRVVEMMEGSLEALEVPPRPVLQQIPISNLHESSILSEDVS
VYTEG*

>AT4G26690

MRGLLRASSLLLCGVILIQLLAAQIHAQSKKPKSPWPTLTGDPPLVIARGGFSGLFPDSSYDAY
NFAILTSVPDAVLWCDVQLTKDALGICFPDLTMRNSSSIEAVYPTRQKSYPVNGVPTSGWFTI
DFSLKDLKDVNLIRGILSRSEKFDGNSNPIMTVQSVSTQMKPSFFWLNVQHDAFYAQHNLSM
SSFLVAASKTVLIDFISSPEVNFFKKIAGRFGRNGPSLVFRFLGQDEFEPTTNRTYGSILSNLTFV
KTFASGILVPKSYILPLDDQQYLLPHTSLVQDAHKAGLEVFVSGFANDIDIAHDYSFDPVSEYL
SFVDNGNFSVDGVLSDFPITASASLDCFSHVGRNATKQVDFLVITKDGASGDYPGCTDLAYK
KAIKDGADVIDCSVQLSSDGTPFCLSSIDLGNSTTVSLTAFRNRSTTVPELGSLGAIYTFSLTWA
EIQTLTPAISNPYRVTSLFRNPKQKNAGKLFSLSDFLSLAKNSTSLSGVLISVENAAYLREEQGL
DVVKAVLDTLTQTGYSNSTATKVMIQSTNSSVLVDFKKQSQYETVYKVEENIRDILDSAIEDI
KKFADAVVIQKLSVFPVAQSFITTQTNVVEKLQKSQLPVYVELFQNEFLSQPYDFFADATVEI
NSYITGAGINGTITEFPFTAARYKRNLCLGRKETIPYMAPAQPGALLTLVSPTAFPPAEAPNPV
FTDADVTEPPLPPVTAKAPTSSPGTPSTNAQAPSGQTRITLSLLLSVFAMVLASLLLL*

>AT5G55480

MINMRDNPTMHVLQASKFLFLALILIQLLSTQLFAQRSKSPWQTLTGDAPLVIARGGFSGLLP
DSSLDAYSFVSQTSVPGAVLWCDVQLTKDAIGLCFPDVKMMNASNIQDVYPKRKTSYLLNG
VPTQDWFTIDFNFKDLTKVILKQGILSRSAAFDGNSYGISTVKDISTQLKPEGFWLNVQHDAF
YAQHNLSMSSFLLSISKTVIIDYLSSPEVNFFRNIGRRFGRNGPKFVFRFLEKDDVEVSTNQTY
GSLAGNLTFLKTFASGVLVPKSYIWPIESQYLLPRTSFVQDAHKAGLEVYASGFGNDFDLAYN
YSFDPLAEYLSFMDNGDFSVDGLLSDFPLTASSAVDCFSHLGSNASSQVDFLVISKNGASGDY
PGCTDLAYTKAIKDGADVIDCSLQMSSDGIPFCLSSINLGESTNVVQSPFRNRSTTVPEIGSLPG
IYSFSLAWSEIQTLRPAIENPYSREFTMFRNPRERSSGKFVSLSDFLNLAKNSSSLTGVLISVEN
ATYLREKQGLDAVKAVLDTLTEAGYSNKTTTTRVMIQSTNSSVLIDFKKQSRYETVYKVEETI
RDILDTAIEDIKKFADAVVISKKSVFPTSESFTTGQTKLVERLQKFQLPVYVEVFRNEFVSQPW
DFFADATVEINSHVTGAGINGTITEFPLTAARYKRNSCLTRKDVPPYMIPVQPAGLLTIVSPAS
LPPAEAPSPVFTDADVTEPPLPPVSARAPTTTPGPQSTGEKSPNGQTRVALSLLLSAFATVFAS
LLLL*

>AT3G20520

MACPRVIFLILITFFILQTAFSSSWQTLSGKPPAVIARGGFSGMFPDSSIQAYQLVNITTSPDVML
WCDLQLTKDGVGICFPNLKLDNGSNVIRIDPHYKERFSVDFTWKELSDVKLAQGVVSRPYIF
DDVSSILAIEEVAKLTASGLWLNIQDSAFYAKHNLSMRNSVVSLSRRLKVNFISSPGISFLKSM
KNSVKPTVTKLIFRFLKQEHIEPFTNQSYGSLAKNLSYIRTFSSGILVPKSYIWPVDSALYLQPH
TSLVTDAHKEGLQVFASEFANDFVIAYNYSYDPTAEYLSFIDNGNFSVDGFLSDFPVTPYRAIN
CFSHVDPKRAKEQAKITIISKNGASGDFPGCTDLAYQRAASDGADILDCNVQMSKDKIPFCMS
SFDLINSTNVIETSFRNLSSVVSEINPRRSGIYTFSLTMSQIQTLKPTISNLEKDSGLFRNPRNNK
AGKFLTLSEFLFLPNRYSSLLGLLIEVENAAYLVEHQGISVVDAVLDELKRATTQQNKTSARTI
LIQSTDKSVLMKFKEKNKMNHDELVYRVDDNIRDVADSAIKDIKNFAGSIVISKKSVFPYKGF
IILEKETNIASKLKSNGLRVYVERFSNECVTHAFDFYDDPTLEIDSFVRDVQIDGIITDFPATTA
RYRKNKCYGEFGLTTTGELITFANPMLLPPAEAPYPALLDSDVTEPPLPEARSQPPASSPSKAE
EKAIEVPFAFIAMAILVCFFISV*

>AT5G58050

MLRFFILFSLFLHSSVAAPKTPAAAAAVPAKKWLTLNGQEPAVVARGGFSGLFPESSISANDL
AIGTSSPGFTMLCNLQMTKDGVGLCLSDIRLDNATTISSVFPKAQKTYKVNGQDLKGWFVID
YDADTIFNKVTLVQNIFSRPSIFDGQMSVSAVEDVLGTKPPKFWLSVQYDAFYMEHKLSPAE
YLRSLRFRGINVISSPEIGFLKSIGMDAGRAKTKLIFEFKDPEAVEPTTNKKYSEIQQNLAAIKA
FASGVLVPKDYIWPIDSAKYLKPATTFVADAHKAGLEVYASGFANDLRTSFNYSYDPSAEYL
QFVDNGQFSVDGVITDFPPTASQSITCFSHQNGNLPKAGHALVITHNGASGDYPGCTDLAYQ

KAIDDGADIIDCSVQMSKDGIAFCHDAADLSASTTARTTFMSRATSVPEIQPTNGIFSFDLTWA
EIQSVKPQIENPFTATGFQRNPANKNAGKFTTLADFLELGKAKAVTGVLINIQNAAYLASKKG
LGVVDVVKSALTNSTLDKQSTQKVLIQSDDSSVLSSFEAVPPYTRVLSIDKEIGDAPKTSIEEIK
KHADAVNLLRTSLITVSQSFATGKTNVVEEMHKANISVYVSVLRNEYIAIAFDYFSDPTIELAT
FIAGRGVDGVITEFPATATRYLRSPCSDLNKDQPYAILPADAGALLTVADKEAQLPAIPPNPPL
DAKDVIDPPLPPVAKLASNGTEGGPPQTPPRSGTVAIAANLSLSLLAMMALGLLYTA*

>AT5G58170

MLRFIIFFSLFIHLCVAAPQTPAAAAAVPAKKWLTLNGQEPAVVARGGFSGLFPESSASANDL
AIGTSSPGLTMLCNLQMTKDGVGLCLSDIILDNATTISSVFPKAQKTYKVNGQDLKGWFVLD
YDADTIFNNVTLVQNIFSRPSIFDGQMSVSAVEDVLGTKPPKFWLSVQYDAFYMEHKLSPAE
YLRSLQFRGINVISSPEIGFLKSIGMDAGRAKTKLIFEFKDPEAVEPTTNKKYSEIQQNLAAIKA
FASGVLVPKDYIWPIDSAKYLKPATTFVADAHKAGLEVYASGFANDLRTSFNYSYDPSAEYL
QFVDNGQFSVDGVITDFPPTASQSITCFSHQNGNLPKAGHALVITHNGASGDYPGCTDLAYQ
KAVDDGADVIDCSVQMSKDGIAFCHDAADLTASTTAMTIFMSRATSVPEIQPTNGIFSFDLTW
AEIQSVKPQIENPFTATGFQRNPANKNAGKFITLADFLDFSKAKAVTGVMINIENAAYLASKK
GLGVVDAVKSALAKSTLDKQSTQKVLIQSDDSSVLASFEAVPPYTRVLSIDKEIGGAPKPSVD
EIKKYAEAVNLLRTSLVTVSQSFTTGKTNVVEEMHKGNISVYVSVLRNEYISVAFDYFSDPTIE
LATFISGSGVDGVITEFPATATRYLKSPCSDLNKEQPYAILPAEAGGLVVVADKEAQPPASAP
NPPLEAKDVIDPPLPPVANLAASNATGGAQSHPPPASGTVANAANLGLSLLAMLALGV*

Oryza sativa

>Os12g120800

MLSYRWILLLSVVVVVAVAAELPLASTTFKTLTGNAPLVIARGGFSGLFPDSSQFAFGFALSA
TSTDTSLWCDVQLTKDGVGFCLRDLLMQNGTTISQVYPGGKQTYRINGVPKTGWFPVDYNM
SLLTNVFLTQALFSRTDKFDFCNFRIFSVTGFMSSIEPSSLWLNVEHDVFYTEHGLNMTNYILS
VQKLGFVKYISSPELGFLETLSGGIDHKVKLVFRFLDKAVSDLSTNKTYDSMLSDLAFIKTIAS
GIMVPKSYIWPVTSDNYIQLPTQIVKDAHDAGLEIYASDFSNDGIFPYNYSYDPLEEYLSFVSN
GGFSVDGVLTDHPLTASEAIGCFTNLNTSRKTDHGNILIISHNGASGDYPDCTDLAYEKAVGD
GADVIDCSIEMTKDGIPICMSSINLYDSTDVQNSKFSSLASVVPEIQTKPGIFTFNLTWEEISTLR
PKITHPYHDFVRNPRYANQGKFFKLSDFLTYAKDKDLSGIMIIMKNAAFMAKSLGFDVVDLV
TTALSNAGYDNMDPTTKNNKEIMIQSKDSAVLVKLKQRLTQCKLVYSLPLKVGDVSDSCVA
DIKKFADAVIVDRESVFAESKGFIIRKSKVVEDVQSAGLAVYVEVFRNEYVSPPVDFFADGTV
EINNYVQLVHVDGFITDFPKTVRRYKMNTCTRQGDGTSTSMKQVPIGDLAQLLDAECSTGG

MLPALAPMPVLNSSDVIEPPLPAAEPKSAAGSSATNACVVGVLAPPPLFSSSREYVLLATLLLL
QML*

>Os03g250500

MYVYEILGLISIIVLVLLSGESNANPAASQQSQLDVNHRKPLQTFRPYNIAHRGSNGEIPEETTA
AYLRAIEEGADFIESDILATKDGHLICFHDVILDATTDIANRTEFANRKRTYEVERQNVTGWF
VVDFTLEELKSLRVKQRYNFRDQQYNGKYQIITFDEYILIALYADRVVGIYPELKNPIFINEHV
KWSDGKKFEDKFVQTLLKYGYKGEYMSEDWLKQPLFIQSFAPSSLIYMSNMTNSPKIFLIDDT
TVRTQDTNQSYYEITSDAYLAFIRKYIVGIGPWKDTIVPPINNYLGPPTDLVARAHALNLQVHP
YTFRNENMFLHFDFHQDPYLEYEYWLGEIGVDGLFTDFTGTLHRFQECTTPYPKNEKNAEAL
LQKINYMLKDSGY*

>Os04g164200

MRGSHVCSLVSSLVFLWLGVAAAQKASSWKTLSGNAPAIIAKGGFSGIFPDSSEFAYQFALIA
SSPDTILYCDVRLTKDGLGICLPDIKMDNCTNIPDFYQQGRKSYLVNGVSTAGWFSVDYNGT
ELGQVSLKQSIFSRSPRFDPSFFPILAVEDIASKFKPPGMWLNVQHDSFYSQFNLSMSNYIFSVS
KRVIVDYISSPEVSFLTKVSGKLSNNTRLVFRFLDESTIEPSTKQTYGSMLKNLTFVKTFASGII
VPKKYIWPVSPDNYLEPHTSVVDDAHKAGLEIYAADFANDFMFSYNHSYDPLAEYLSFIDNG
AFSVDGVLTDFPVTPSEAIGCFTNLKKSKTDHGKPLIISHNGASGDYPACTDLAYQKAVDDGA
DVIDCPVQLTKDGIPICMSSINLMDDTTVAKSQFASQTAVIKDIESVLGVFTFNLTWDDIVKNL
RPKISTPFSSFKLDRNPRYRNAGNFMRLSDFLDFTKDKDLSGIMISVEHAAFVAEELGFDMVD
SVIKTLDAAGYSNQTAQKVMIQSSNSSVLVKFKQQTKYDLVYMINEEVKDAAPSSLAAIKKF
ADAVSVEGNSIFPENRHFTTYQTNLVESLQNAGLPVYVYTLMNEFASQPYDFFSDATAQINA
YVQGAGVNGVITDFPATARRYKLNTCMHMGNNTPSFMAPARPGDLLQIISKPAQPPAMSPMP
LLTGSDVAEPPLPPARTAQAPSLASRMQAHAAIVVTLAMLLACHPLV*

>Os11g084800

MRRLLGIGRRRQQQPPPLPLFPPPATKRASPPSSSSSSSALLRRILPTNRLLRLLLLLAALSLIPP
AFFHFRLRRFHRMRERRCGWIASPPMVCAHGGDSTNAFPNSMDAFRLALDARVDCVEVDVS
RSSDGALFVLHDRDLQRMSGNSTAKVGYWSSDEIKALSTRFQLSKKVQNQEVPKAQDVLALI
SQSVRQVILDVKVGPPSFEKDLAEDVLSIIGRTQCKNCLVWAKSDNVGRDVIKLSKDITVGYI
VMVDKSTGRTTELVRITGSKVAGVYHRLIHEKLMKVMHRNDKKVYAWTVDDANSMKRML
YEHVDAIVTSNPSLLQQLMQETRTECMEDGFALP*

>Os02g184900

MRAMAGHGERIPTPNIPHSRFLPSFLPFSNDPHPTLDPFPLPPPCYKLGSAPASSRRVASRLVVA
LHCAHDPPIRAQLGHPPNIMAQLKAARVADVPMALDVLAAGAPATSAILAEVDAAGARSAA
AGGGGGGGGQRFAVIGHRGKGMNALASPDRRMQEVKENSLRSFNEAARFPVDYVEFDVQV
TKDGCPVIFHDNFIFTKEDGKILDKRVTDLQLEDFLLYGPQNEQGKGGKPLLRKLKDGRIVN
WNVQSDDPLCTLQEAFEKVNPRLGFNIELKFDDNLEYQEEELTCILQAILKVVFEYAKDRPIIF
SSFQPDAAQVMRKLQSTYPVYFLTNGGTEIYADVRRNSLEEAIKLCLASGMQGIVSEARGIFR
HPAAVPKIKEANLSLLTYGTLNNVPEAVYMQHLMGVNGVIVDLVQEITEAVSELITVPEPDLN
ADNLSNGAAKDAATPHFSQCEISFLLRLIPELVQ*

>Os02g226400

MGRGSHGCSVLGSSLLLLFCLGSAAAQKASTWKTLSGNPPAIIAKGGFSGLFPDSSDFAYGFV
AAASSPDTALWCDVQLTKDGAGICLPDIRMDNCTNIANVYPKGKKTYSVNGVSTPGWFSVD
YDSTGLSKVNLVQSLFSRVPYYDGTLPILPVESVFANYKAPAVWLNVQHDSFYSQFNLSMRS
YILSVSKQYIADYISSPEVNFLTSLSGRVNKKTKLVFRLLNELAVEPSTNQTYGSMLKNLTFIK
TFASGILVPKNYIWPVTQDNYLQPSTSVVGDAHKAGLEVYAADFANDFLLSYNYSYDPLTEY
LNFIDNGAFSVDGVLTDFPITPSEAIGCFSNLNNSKTDNAKPLIISHNGASGDYPDCTDLAYQK
AVTDGADVIDCPVQVTKDGIPICMSSIDLMDVTTVSTSQFSSQTTVIKDIKNGAGVYSFNLTW
DDIAKNLKPKISNPMTTFDVYRNPRNKNAGSFMRLSDFLAFAKGKELSGVMISIEHAAFMAE
KLGFGVVDAVIKALDDSGYSKQTAQKVMIQSTNSSVLVKFKEQTKYNLVYMLEEDVRDAAP
SSLADIKKFANAVSVRTTSIYPESKHYLINQTSHIVQTLQSAGLPVYVYVLMNEFVSQPNDFFA
DATTQINTYVQKKGAGVDGIITDFPATVHRYRLSPCTSKESNLPTFMLPVQPGGLSGTIIDPAA
QPPAMAPMPLLTDSDVAESPLPPVKNVTAPAPGASRAIKMRTDASIIVALLVLCASLII*

>Os01g335600

MRPSLRTIYFLLLLLPHVVFSRPLFPLPSKSNGIEKRPLQTFRPYNIAHRGSNGEIPEETAAAYL
RAIEEGADFIETDILASKDGALICFHDVTLDATTDVASRKEFSNRRRTYEVEWFNATGWFVVD
FTLEELKTLKVNQRYPFRDQQYNGKFSIITFEEFISIALDASRTVGIYPEMKDPVFINKHVKWD
GGKKFEDKFVDTLLKYGYKGQYMSENWLKQPLFIQSFAPTSLVHASKLTDSPKIFLIDDFSVR
TQDTNQSYWDITSDDYLAYISNYVVGLGPWKDTVVPAAKNYTMAPTDLVARAHAHNLQVH
PYTYRNENQFLHLNFHQDPYAEYDFWINSMGVDGLFTDFTGSLHRYQELVAPHAKDETANS
LLVKIAQMISQYEGF*

>Os08g232100

MALPAATARAAALCRIAACASASSAAAAATGGRRGAEKLPFSLAERGMVVGGHRGMGMNA
VGAPPGARIEAVRERENTLLSFGRAAAHAAVAFVEFDVQVTKDGCPIIFHDDFILTQETDAVY

AKRVTDLLLEEFLSYGPQKNSHEISKPLLRRTSDGRVVNWSAKDDDSLCTLQEVFERVSPRLG
FNIELKFDDDIFYERSQLDRALQAVLQVVSQYASNRPVFFSTFHPDAARIMRELQSLYPVLFLT
EGGTAQHKDSRRNSLDEAIRVCLEYELHGLVSEVRGVLKNPSAVLRAKESNLALLTYGQLNN
VWEAVYIQYLMGVNGVIVDLVEEISNAVADFSKPVLNQSMLGSGVDLVGAKHQAFSQQQLG
FLLWLIPELIQQPH*

>Os09g061800

MLSRITGSLPMVRSYCFCLLLLSTIVAVTVAVAAEELPPAPTTFKTLNGNAPLVIAKGGFSGVF
PDSSEYAFAFASSLHISLWCDVQLTKDGVGICLRDLLMQNCTDITEIYPEGMKAYLINGAQKT
GWLPVDYNMASLRNVTLTQSIYSRTPRFDSNFDILSVTGFISLIKPSSTWLNVEHDIFYREHGL
NMTNYILSIQKLGSVKYISSPELGFLQSLSGGINREVNLVFCFLDKALSDPSTNKTYNYMLSNL
TFIKTIASGIMVPKNYIWPVTSDNYIQLHTQIVQEAHNAGLEIYASDFSNDGIFPYNYSYDPLGE
YLSFVSDGGFSVDGVLTDFPLTASEAIDCFSNLNTSRKTDHGNPLIISHNGGSGDYPGCTDLAY
ENAVRDGADVIDCSIQMTKDGIPICMSSIDLLATTDVQQSKFCSLLSVIPEIQSKKGIFTFNLTW
DDINILRPKISSPLSDYVMLRNPRYTNHGKFLKLSEFLTS*

>Os04g109400

MVAAAAAAAMVVIGHRGKGMNALGSADPRLREVKENSLRSFHAAARVAGVSYVEFDVQV
TKDGYPVIFHDDFIFTEQDGEICGRRVTDLRLDEFLSYGPQKDQSKAGKPLFRKLNDGRVLR
WDVQSDDALCTLQEALDGVDRRVGFNVELKFDDDVVYREMELTGILQAILKVVFEHAKERP
IFFSSFQPDAARIMRKLQDRYPVYFLTKGGTQVFADERRNSLEAAVKLCVAGSLRGIVSEARA
VLRQPSAIGRIKEAGLSLLTYGQLNNVPKAVYLQQLMGVDGVIVDLVAEIAAAVSEFAAAAA
AAVPVPERDSSSSYMDGGGDVGLLEMTSPAARTTASFSRREDVSFLLRLTPELVQ*

>Os02g068400

MGERYPHMFLILLLFHGAHAALKDPVQKWQTLGGQRPLVIARGGFSGLFPDSSQFSYQFAMS
SSLHDVVLYCDLQLSSDGLGFCKTGLTLENSTLIAEAFPKRAKTYKVNGEEIHGWFALDFTAD
ELYQNVTLIQDIFSRPSTFDGAMGMFTLEDLVGLKPPHLWVNVEYALFLQEHKLSAEDYILGL
PKDFSVAYISSPEIGFLKNVGAKLKKSNTKLIFRFLREDVAEHTTKKTYGEILKDLKSVKAFAS
GILVPKEYIWPLGKDQYLRLPTSLVKDAHALGLEVFASGFANDVSMSYNYSFDPSAEYLQYI
GNANFSVDGVITDFPPTASGAVACLANTKGNPLPPPGGDGGRPLIITHNGASGVYPGSTDLAY
QQAVKDGADIIDCAVRMSKDGVAFCQPSADLSTSTTASTSFMTKISTVSEIQNKSGIFSFDLTW
SEIQTLKPDLLGPYTQAGLKRNPAAKNAGKFVTLPEFLDLAKATNVSGIMVEMEHASFLAKR
GLGLVDAVSGALANASYDKEGGHLPELMVVSDDTSVLAAFKKFPAFRRVLMVDETISDASA
PSVEEIKQFATAVTVGRGSIAQVNGFFLTRFTDVADRMHAANLTVYVGVLRNEFMNLGFDY

WADPIIEIATYAFHVMADGLITEYPATAATYFRSPCSDLSLNLSYAILPADAGALVHLAAPGA
LPPALPPAPVLEPGDVLDPPLPPVAIASPPEEAATAAKPTDSSSSAAAARSSAGAGGCRVLAAA
VASLFLILLPSHGFH*

>Os07g219900

MGSFKLALFEPNGLNNPRPNTHGWRWWCLSGARRAAARPLVGGGGAGGTPKAPLQTSRPF
NIAHRGSNGELPEETAAAYMRAIDEGADFIEADVTATKDGHLVCFHDTTLDATTDVADHPEF
ASRRRTLEVQWTNVTGFFITDFTLAELKTLRSKQRYAFRDRSYNGGESSRIITFDEFIDTAAGA
ASRVVGIYPEIKNPVFVNRQVRWRDGKKFEDKFVAALKRRGYGGRYMSPAWAARPVFIQSF
APTSLVYAAGLTDSPMVLLVDDTTVRTEDTSQSYDEVTSDEHLDYMREYVVGVGPWKDTV
VPPTTDNKLAAPTDLVARAHARGLQVHPYTYRNENQFLHFNFRQDPYAEYDYWINDVGDLF
TDFPASLRRYQEWTTAGRKG*

>Os08g203700

MTTSSWGGGGGGGGGAVRLWCCGLLLMLLSGGGGGGGAAAQRPPAYKTLSGNAPIVIAEG
GFSGVFPDSSKNAYVFALSSTSGDTVLWCNVQLTKDGVGICLRDLLMDNCTSISQAYRAGKK
AYLVNGEEKKGWFPIDYTMSSLQSVILTQAIWSRTDKFDFAFLPILPVTNVIDLAKPSSVWLNI
EHDIFYRQHGLNMTKYILSIPKGGSVQYISSPELGFLQSISGRVNRKTKLVFRFLDATSSDPSSN
QTYGSLLSNLTFIKTVASGIMVPKEYIWPVTTNNYIQPAKSIVRDAHSAGLEIYASDFANDRIIP
YNYSYDPLEEYLHFVGSDNFSVDGVLSEFPLTAAAAIGCFTNLNVSSKTDHGSPLIISHNGASG
DYPDCTDLAYQKAVDDGADVIDCSIQMTSDGVPVCMSSINLFETTNVQRTPFSNRASIFKDIQ
PTPGIFTFNLTWADISSSDLRPKISSPESIYYLVRNPVHKNAGNFFRLSDFLTFAKDKDLSGIMIII
KNAVFMANSLGFDVVDSVTKALSDAGYNNQTTKAKEVMIQSEDSAVLVNLKQLETKYKLV
YTLPSTIGDASASSLVDVKKFADAVIVDRESIFPESQGFIMKETNLVKDLRSAGLAIYAQVFRN
EFVSPPWDFFSDVTVEINSYVQSVNIDGIITDFPKTVRRYKMNSCTGLGVNMPSYMNPAEIGG
LAQLLNGSQAQPPALAPMPVLNSSDVTEPPFPSAAPKNAPGGAANGSTPAPGASPSGSQAAA
VMRAGILPMVTALFASLLI*
