# Supplementary material for: Genome-wide identification of GDPD gene family in foxtail millet (Setaria italica L.) and functional characterization of SiGDPD14 under low phosphorus stress
Source: Front Plant Sci. 2025 Jun 18;16:1586547. doi: 10.3389/fpls.2025.1586547 (PMC12213840; doi:10.3389/fpls.2025.1586547)
Supplement: Supplementary file 2 [file DataSheet2.docx]

The CDS of SiGDPD gene family in Setaria italica

>SiGDPD1 SETIT_017495mg

ATGGCCCAGCTCAAGGCCGCGCGCGTCGCCGACGTGCCCAACCTGGACGTGGTGGCC CCCGGCCTGGTGGTCGCGGCCGGCGAGTCGGACGCCGCCGCGATCGCGGCGGCGGGG AAGCGCGGCCCGGCGGGCGGCGGCGGCGGCCGGTTCTCCGTGATCGGGCACCGCGGG AAGGGGATGAACGCGCTGGCGTCCGCGGACCGGCGGCTGCAGGAGGTCCGCGAGAA CACCGTCCGCTCCTTCAACGACGCCGCGCGCTTCCCCGTCGACTACGTCGAGTTCGAC GTCCAGGTCACCAAGGATGGGTGCCCAATCATCTTCCATGACAACTTCATCTACACTG AAGAAGATGGTAAAATTTCACAGAAGCGTGTCACTGATCTTCAGTTGGAAGACTTCG TCCAGTATGGACCACAAAATGAGAAGGGGAAGATTGGGAAGCCCCTGCTTCGCAAAA TGAAGGACGGTAGAATCATCAACTGGAATGTGCAGTCAGAGGATGCTCTTTGCACGC TTCAAGAAGCATTCGAGAAGGTCAACACTAGATTGGGCTTCAACGTTGAGCTGAAAT TCGATGACAATCTTGAATACCAGGAGGAAGAGCTAACTCGCATCCTCCAGGCCATCC TGAAGGTGATCTTTGAGCACGCCAAAGATAGGCCTATACTTTTCTCTAGCTTCCAGCC CGATGCTGCGCTGCTCATGCGAAAATTGCAGAGCAAATACCCTGTCTACTTTTTGACG AACGGAGGGACAGAGATTTACATTGACGTGAGGAGGAACTCGTTGGAGGAGGCCAT CAAGCTATGCCTTAGCAGTGGCCTGCAAGGGATAGTTTCTGAGGCCCGTGGAATATTC AGGCACCCTGCTGCCATACCAAAGATCAAAGAGGCTAACCTCTCCCTGCTGACCTAC GGAACATTGAATAACGTGCCAGAAGCGGTGTACATGCAGCACCTGATGGGGGTGAAC GGGGTGATCGTCGACCTGGTGCCAGAGATCACTGAGGCCGTCTCCGAGCTCATCGCC CTGCCGGAGCCTGACCCGGAAGTGGAGAGCTTGAGCAACAACCAGGCTGCTAAAGGC ACTGCAACACCAAATTTCTCGCAACGCGAGATCTCATTCCTGCTGAGGCTTATCCCTG AGCTTGTCCAATAA

>SiGDPD2 SETIT_020031mg

ATGGGAATGTACACGCTTGATGATATTGTTGAGCTCCGCCCCCCTCAAATATGGCTTA ATGTACAGGACACCAGGCCGCTGATCATCACCCACAATGGGGCTGGCGGTGTCTTTG CTGGGAGCACAGATCTTGCCTACCAGGAGGCGATCAAAGATGCCGCCGACATCATAG ATTGCTCGGTTCAGATGTCAAAAGACGGAGTGGCCTTCTGCATGCACTCTGCCGATCT CCCCCCCCCCCAAACCCACGAAAAAAACCGATCAAGCATTCTTGTGCGGGATGCACG CGTGCAGCATGCCTCATATCTCGCCAAGAGAGGTCTCGGTGTGGTGGAGTCGGTGTCC AGCGCGCTAACCAAGGCCGGCTACGACAAGGAGACCAAGCAACAGGTGTTCATCCA GTCCGATGACTCGTCGGTGCTCTCGGCGTTCAAGAAGTTCCCGGCGTTCAAGCGGGTG CTCAACCTCGAGATGGAGTTTAGCGGCGCCTCCCAGCCGTCGCTGGACGACATCAAG AAATTCGCCGACGGGGTGAGGATCCACCGGAGCTCGGTGGCGCAGATCACCGGGTAC TTTATGACGCGGTTCACCGACACGGTCGGCAGCCTGCAGGCCGCCAACCTCACCGTGT TCATCGGCGTGCTCAAGAACGAGTTCATGAACCTTGGCTTCGACTACTTCGCTGACCC GACCGTCGAGATCGTCACCTACTCCTCGGCGGTGATGGCAGACGGGCTCATCACCGA CTACCCTGCCACTGCACTGCAGCTTCATACTTCAGTGAGGAGTCCATGCAGTGACATG AGCCTGAACCTGAGCTACTCGATCCTGCCAGCGCAACCCGGTGCTCTGGTCCACCTGG CAGCCCCCGGGGCGCTAGCACCGGCGGCAGGGCCGGCGCCGTTGCTGGAACCCAAGG ACGTCGTGGACCCGCCGCTGCCTCCTGTCAAAGCTGTGATCGCCGCCGACGCGCCGG

CGCCGAAGGGGACCGCTGACAACACCTCCTCGGCGGCCAGCTTCAACGCCGGGAAGA ACAGCCTCTTGGGAGCTGGCATCGTTGCCCTCTTGTCCCTGAGCTTCCTGCACTGA

- SiGDPD3 SETIT_032348mg

ATGGATGCTGGTTCAGTTGAGGCACACCCGGCTGGGGTGGTCGCGGCCGTGTTGGTGG CGGCGGCGGCCGTCGCGTCCAGCGTCGTCGCGGCACGGCCGCTGGTCGGGGAAGGTG GAGGCGGGGCGTGGGGGGACAGCAGGGCGCCGCTGCAGACGTCGCGGCCCTTCAAC ATCGCGCACCGGGGCTCCAACGGCGAGTTTCCCGAGGAGACGGCGGCGGCGTACGCG CGCGCCATCGACGAGGGCGCCGACTTCATCGAGGCGGACATCGAGGCCACCAGGGAC GGCCACCTCGTCTGCTTCCACGACACCACGCTCGACGACGCCACCGACGTCGCCGAC CACCCGGAGTTCGCCGGACGCCGCCGCACGCTCGAGGTGCAGTGGGCCAACGTCACC GGATACTTCATCACTGATTTCACGCTGGCGGAGCTGAAGACGCTGAGGGCGAAGCAGC GATGGGACTTCCGCGACAAGTCCCACGACGGCATTTCGCCGATCATCACGTTCGAGGA GTTCATCGACATCGCGCTGAACGCCAAGAGGGTGGTGGGGATCTACCCGGAGATGAAG AACCCGGCGTTCATGAACAAGCACGTCCGTTGGGCGGACGGGAAGAGGTACGAGGAT AAGTTCGTCGCGACGCTCAAGAAGTACGGGTACGGCGGCAGGTACATGTCGCCGGCG TGGCGCGCGAAGCCGGTGTTCGTCCAGTCCTTCGCGCCGACTTCTCTGGTCCGCGCCG CCGGCCTCACCGACTCGCCGCTCATCTTCCTCATCGACGACGTGACCGTCCGGACCGA GGACACCAACCAGTCCTACGACGAGATCACCTCCGGCGAGTACCTGGACTACATGAGC AAGTACGTGGTCGGCATCGGGCCCTGGAAGGACACGGTGGTGCCGCCGACCAAGGAC AACCGGCTGGCGACGCCGACGGACCTCGTCGCCATGGCGCACGCGCGAGGGCTGCAG GTGCACCCTTACACGTACCGCAACGAGAACAAGTTCCTGCACTTCAACTTCAGGCAGG ACCCCTACGCCGAGTACGACTACTGGATCAACGACGTTGGCGTCGACGGGCTCTTCAC CGACTTCCCGGCGAGCCTCCGCCGGTTCCAGGAGTGGACGGCCAAGAAGAAAGACTG A

>SiGDPD4 SETIT_000875mg

ATGCGCCCATCTCGCTGTTGTGTTCCCCTTTTTCTTCTGCTACTATCACATTTGGCATTTG CTAGACCTCTCTTTCCACTACCAAGTAAGACGAAGAACGAAGAGAAAAGGCCCATAC AGACATTTCGACCATATAATATTGCTCATCGTGGCTCAAACGGGGAAATTCCTGAAGAA ACAGCTGTTGCATACCTGAGGGCTATTGAAGAAGGTGCAGATTTTATAGAGACTGACAT CCTTGCTAGCAAAGATGGGACCTTGATCTGTTTTCATGATGTAACACTTGATGAGACAA CTGATGTTGC541CGAGCATAAGGAGTTTGCCAATCGAAGAAGAACCTATGAAGTGGAG TGGTCTAATGTTACTGGATGGTTTGTAGTGGACTTCACCCTGGAGGAACTTAAAACACT TAAAGTGAAGCAGAGATACTCATTCAGAGATCAACAGTACAATGGTATGTTTTCAATCA TCACGTTTGAAGAATTCATTTCAATTGCCCTAGATGCGGATAGAACTGTTGGCATATACC CAGAGATTAAAGATCCAGTTTTTATCAACAAGCATGTCAAGTGGGCTGATGGAAAGAA GTTTGAGGACAAATTTGTGGATACCTTGCTTAAATATGGATACAAAGGTCAATACATGT CAGAGAATTGGCTAAAGCAGCCATTGTTTATACAATCCTTTGCTCCCACCTCCATTATAT ATGTATCCAAATTGATTGACTCTCCCAAGGTATTTTTGATTGATGATATAACTGTGAGAA CACAAGACACGAACCAGTCATACTGGGAAATAACTTCAGATGACTACCTTGCATATATT GGGAAATACGTTGTCGGCCTTGGTCCATGGAAAGATACCATTGTTCCAGCTGCAGGAA ACTACTTGATGCCACCAAGTGATCTTGTTGCACGGGCACATGCTCATAATCTCCAGCAT

TCTGTTGTTGTGCAGGTGCATCCATACACTTACAGGAATGAGAACCAGTTCCTGCACTT CAACTTCCATCAGGATCCCTACGCTGAATATGATTTCTGGATAAAGAATGTGGGGGTTG ATGGACTGTTTACAGATTTCACTGGAACCCTTCACCAGTACCAAGAATTGACATCCCCG CACCGGAAGGATGAAACTGCAAACAGCCTCCTAGTAAAAATTAGTCAAATGATCTCAG CATATGAAGGTCTCTGA

- SiGDPD5 SETIT_014004mg

ATGGGTCCGGACGCCAGCCAGCCGTGCCAAATCAGCAAGCGAGAGCGACGCGCACGC GCTCGGAGCTTTGGGGTCAAAATGGCGCTCCTCCCGGCGGCGACCCGCGCGGCGGCG GCGGGGCTCTGCCGGATCGCGGCGAGCGCGTCCTCCTCGCCGCCGTCGTCGGCTCCGG CGAGGAGGGAGCGGGAGCTGCCGCTCGCGCTGCTGGCGGAGCGGGGGAAGATGGTG GTGGGTGGCCACCGCGGGATGGGGATGAACGCGGTGGGGGCTCCGCCGGGGGCCCGC GTCGGGGCCGCCAGGGAGCGGGAGAACACGCTGCTCTCCTTCGGCCGCGCCGCCGCG CACGCCGCCGTCGCCTTCGTCGAGTTCGATGTCCAGGTCACAAAAGATGGCTGCCCAA TAATCTTCCATGATGACTTCATCTTAACACAAGGAATTGGGGCTGTATATGAAAGGCGT GTAACTGATCTTCTCTTGGAAGAATTCCTCTCATATGGGCCACAAAAAGAATCTCGCAA GGTCTCTAAGCCTTTACTTAGACGGACAGGAGATGGTAGAGTCCTTAATTGGAGTACA GAAGAAGATGATTCCCTTTGCACATTGCAAGAGGTCTTTGAATGTGTCAGTCCTCATCT GGGATTTAACATTGAGCTAAAGTTCGATGACAACGTTCTCCATCACAAGAAAGACCTT GAACGTGCTCTTCAAGCTATATTGCAGGTTGTTTTTCAGAATGCTAGGGATAGACCAGT TTTTTTCTCATCTTTCCATCCTGATGCGGCTATGATGACACGGGAGCTCCAAAGTTTATA TCCTGTACTTTTCTTAACAGAAGGAGGGACATCCAAACACCATGATTCAAGAAGGAAC TCACTGAACAATGCCATTCAGGTATGCTTAGAGCATGATTTGCATGGGATCGTGTCAGA CGTCAGAGGCATTCTCAAGAACCCTGCTGCAGTCGTCAGAGCACAAGAATCCAACCTT GCACTTCTCACTTACGGGCAGCTCAATAACGTGTGGGAAGCAGTCTATGTCCAGTACCT GATGGGCATAAACGGCGTCATCGTTGATCAGGTGGAGGAGATCTCAAATGCTGTGGCG GGGTTCAGTAAACCAGACCTCAACCGGAGCAGTGCTGGTGTGGACGGAGCAACACAT CAGGCCTTCTCGCAGCAGCAGCTTGGGTTCTTGCTCCGGCTTATACCTGAATTGATTGA GCAGCGACATTGA

- SiGDPD6 SETIT_010283mg

ATGGCTCAGCGCAAGGCCGCGCGCGCTGCCGCCGACGCCGCCGTCCCGCCCGCCATG CCGGAGCCGGACATCGCCGCGGAGCTCCACGCCCTAGTGGCCTCCTCCACCTTCCCCG CGCCGTGCATCGGAGGTGCCGCGGCGGCGAGGGACGACGCCGGGCGGAGGCGGCCG CCGCTCGTGGTGATCGGGCACCGCGGGAAGGGGATGAACGCGCTGGCGTCGCCGGAC CCGCGGGTGCGCGGCGACGTCAGGGAGAACACGCTCCGCTCCCTCAACGCCGCCGCC GCCAGCCACCCCACCGTCGCCTACGTCGAGTTCGACGTCCAGGTCACCAAGGATGGCT GCCCGGTCATCTTCCACGACAACTTCATCTACACCCAAGAAAATGGCGAGATCTCAGG GAGGCGCGTCACCGAGCTTCACCTCGACGAGTTCCTCTCCTACGGGCCCCAGAGGAA CCAGGAGAAGGCTGGGAAGCCGCTGCTCCGGAAGCTCAAGGACGGGAGGATCCTCA AGTGGGACGTGCAGAAAGAAGACGCGCTCTGCACGCTGCGGGAGGCGTTCGAGGGC GTCGACCGCCGCGTCGGGTTCAACGTCGAGCTCAAGTTCGACGACGACCTCGCGTAC ACGGAGGAGGCCCTCACCGGTGTCCTGCAAGCCGTCCTCAAGGTTGTCTTCGAGCAC GCCGACGGCAGGCCCGTCATCTTCTCCAGCTTCAAGCCCGACGCCGCGCTGCTCATCC

GGAAGCTGCAAGACAAGTACCCTGTGTACTTCCTGACGAACGGAGGGACGCAGATCT ACGCGGACCCGAGGCGGAACTCGCTGGAGGAGGCCGTCAGGCTGTGCGTCGCCGGTA GCCTACAGGGGATCGTGTCGGAGGTCCGGGCGATACTCCGGCAGCCGGCGGCGGCGG CCAAGATCAAGGAGGCCGGGCTGTCGCTCATGACCTACGGCCAGCTCAACAACGTGC CGGAGGTGGTGTTCGTGCAGCACCTGCTGGGGGTGGACGGCGTCATCGTCGACCTGG TGCGGGAGATCGCCGAGGCCGTGTCAGCGTTCTCGGCCGCCGCGCGGGAGCCGAGCC CCGAAAGCTGCGGGGAGGTTGAGAGGCTAGAGACGGCGGCGGCGGGGACGCCGAGC TTCTCCCCGCGGGAGATGTCCTTCCTGCTCAGGCTCATCCCCGAGCTGGTGCAGTAG

- SiGDPD7 SETIT_0280281mg

ATGAGGGGGCTCCTCGGGCGCCGGCGGCAGCCGCCGCCGCTCCCCCTCTTCCCCGCCG CCAAGCGCTCCTCCGCGCCGGCGAGCCTCCTCTTCGCGCGCCTCAGCCGTCTCCTCCC GGCGAGCCGCCTCCTCCGCCTGCTCCTCCTCCTCGCGCTCCTCTCCCTCGTCCCACCGG CCTTCTTCCACCTCCGCCTCCGCCGCTTCCACCGCATGCGGGAGAGGAAGTGCGGGTG GATCACGAGCCCGCCCATGGTGTGCGCGCACGGCGGCGATTCCACCAACGCGTTCCCC AACTCGATGGAAGCATTTCGGATGGCTCTGGACGCACGGGTCGACTGCATGGAGGTAG ACGTCTCGAGGTCCTCAGATGGCGTGCTCTTCGCGCTGCATGACAGGGATCTGCAAAA AATGTCTGGTAATTCCACAGCCAAAGTTGGCCACTGGAGCACAGATGAGATAAAAGCA CTAAGGTTTCAGCTATCGAAAAGAGTCCAAAACGAGGAAGTTCCTAAAGCAGAAGAT GCTCTGGCAGTGATATCACGATCAGTTAGGCAGGTGATTCTGGATGTGAAAGTTGGCCC ACCTTCATTTGACAAAGGTTTAGCTGAAGATGTATTGTCTCTTCTGAAGAGAACAAATT GCAAAAATTGTCTTGTGTGGGCAAAAACTGATAACCTAGCAAGAGATATAATCAAGTT GTCTGAAGATGTTGTGGTTGGTTATATTGTTATGGTTGATAAGTCCACCGGTAGAAAAA CCGAGTTTGTGCGGCTCGAGGGGGCTAAAGTTGCTGGTGTATATCATCCTTTGATCCAT GAGAAAGTTGTGAAGGTCATGCGCAGACATGGTAAGAAAGTCTTTGCATGGACTGTTG ACGATAACAAGTCCATGGAGAAGATGCTGCATGAGCATGTTGATGCCATTGTGACCAG CAACCCTTCCCTCCTGCAGCAGCTTATGCATGAGACAAGAACTGAGTGCCTGGAAGAT GGTTTTGCCTTGCCATAA

- SiGDPD8 SETIT_036072mg

ATGGCTTCCTCCTGGTACACCTCTATCATTGTCCTTGTGCTGCTTTTTGGGGGATCCAAA GCCAACCCTGCTGCAACATCCCACGGCCAGCTGGATGTAAACCACAAGAAGCCACTAC AGACATTCAGACCTCACAACATTGCCCACAGGGGCTCAAATGGCGAATTGCCCGAAGA GACAGCAGCGGCCTACCTGAGAGCTATTGAAGAGGGTGCAGACTTCATCGAGACCGAT ATACTCGCATCAAAGGATGGACATCTGATCTGTTTTCATGATGTAACGTTGGATGCAAC AACCGATGTTGCGAACCGGAGGGAGTTTGCCAACAGGAAGAGAACCTATGAAGTCGA AGGAGCAAAAATGACCGGATGGTTTGTTGTGGACTTTACTCTTAAAGAGCTTAAATCA CTGAGGGTGAAACAACGGTACAGTTTCAGGGACCAACGGTACAATGGAAAATACCAA ATAATTACATTTGAGGAGTATATCTTGATTGCGCTTTACGCCGACAGGATTGTCGGGATA TATCCTGAGATCAAAAATCCTGTTTTCATCAACCAACATGTCAAGTGGTCAAACGGAA AGAATTTTGAGGATAAGTTTGTCGAGATACTACTGAAATATGGCTATAAAGGTGAATATA TGTCTGAAGATTGGTTCAAGCAACCGCTGTTCATACAATCCTTTGCTCCAACTTCACTC ATTTACATCTCGAATATGACAAACTCTCCTAAAGTGTTCCTAATTGATGACACGACAGTT CGAACTCAAGACACCAATCAGTCATACTATGAGATAACTTCAAATGCCTACTTTGCATT

CATAAGAAATTATGTTATTGGAATTGGTCCATGGAAGGATACAATTGTTCCTCCAAAGA ATAACTATTTAGGACAGCCGACCGATCTCGTCGCACGAGCACATGCTCTTAATCTTCAG GTGCATCCTTACACATTCAGAAATGAGAATTCATACTTGCACTTCAACTTCAATCAAGA CCCTTATGTCGAATATGAGTATTGGCTCAATGAGATCGGTGTCGATGGGCTATTCACTGA TTTTACTGGTAGTTTGCACAAGTACCAAGAATGGACTACACCATACCCAAAGAAGGAG AAGAATGCGGAAGCACTGTTGCATGAGATCGCCAACATGTTGAAGGCTGATGGATACT GA

- SiGDPD9 SETIT_016242mg

ATGGGGAGGGGCAGCCGCGCCTGCTCCGTCCTTGGCTCCGCCCTGCTCCTGCTGCTGG TCTCGCTCGGCTCCGCCGCCGCGCAGAAGGGCTCCACCTGGAAGACCCTGAGTGGCA AGGCTCCAGTGATCATTGCTAAGGGCGGGTTCTCGGGCCTTTTCCCAGATTCCAGTGAT CTTGCTTATCAGTTTGTTCCTATTGCTAGCTCTCCTGACACAGCCCTGCTGTGTGATGTT CGGTTGACCAAAGATGGTGCTGGAATCTGCCTTCCCAACATAAAGATGGACAACTGTA CGTTCATATCTGATGTTTTCCCACAGGGTAAGAGCACTTACAATGTTAATGGTGTATCTA CGACGGGGTGGTTCTCCGTGGACTTCACAAGCACTGATCTGCAAAACGTAACCTTGAG GCAATCGATCTTTTCCCGTCCATCTTACTTTGATGGTTCCATGCAAATAGTTCCTGTTGA AGCTGTTCTGTCCGTATTTAAGGCCCCTGCTGTTTGGTTAAATGTGCAGCATGACAGTT TCTACAGCCAGTTTAAGCTCAGCATGAGAAGCTATATCCTGTCTCTGTCAAAACAATAC ATTGCCGACTACATCTCATCGCCTGAAGTGAACTTCCTCACCAGCATATCTGGAAGAGT TAGCAAAAGGACAAAGCTTGTGTTCCGCTTTCTTGATGAACGCTCCATTGAGCCATCTA CAAACCAGACATATGGTTCAATGTTGAAAAATCTGACTTTCGTCAAAACTTTTGCTTCT GGAATACTTGTCCCCAAAAACTATATTTGGCCTGTGACACCAGACAATTATCTGCTCTC CTATACATCAGTCGCTGCTGATGCTCATAAAGCAGGGCTAGAAATCTATGCTGCTGATTT TGCAAATGACTTTACTATCAGCTACAACTACAGCTATGATCCATTAGCAGAATACCTTTA CTTCATTGATAATGATGCCTTCTCTGTTGATGGTGTATTGACTGATTTCCCTATCACTCCT TCAGAGGCAGTTGGCTGCTTTAGTAACCTGAACAACAGCAAGACAGATCATGCTAAGC CTCTAGTTATCTCTCATAATGGTGCCAGTGGTGACTACCCAGACTGCACTGATCAAGCT TATCAGAAGGCAGTTGATGATGGTGCAGATGTCATTGACTGTCCTGTTCAAGTGACCAA AGATGGCATACCAATATGCATGAGTTCAATTGACCTAATTGATGTTACTAATGTTGCAAA ATCAGAATTTGCTTCGCAAACAACTACCATAAATGACCTGAAAGCTGGTCCTGGAGTC TTTACCTTCAACCTCACTTGGGATGATATTTCGAAGAACCTGAAGCCCATGATATCAAG CCCAATGAACAAATACCTACTATTCAGAAATCCAAGAAACAAGAATGCTGGGAATTTCA TGAGGCTATCAGACTTTTTGGCCTTTGCAAAAGACAAGGATTTGTCGGGAATCATGATC ACGGTAGAGAATGCTGCATTCATGGCAGAGAAACTTGGATTTGGTGTGGTAGATGCAG TGATCAAAGCCCTTGATGACTCTGGTTACAACAAACAGACCGCCCAGAAAGTTATGAT TCAGTCAACCAACAGTTCGGTTCTAGTGAAGTTCAAGCAGGAAACCAAGTACGACCTT GTCTACATGATCGAAGAATCTGTCAGAGATGCTGCACCTTCCTCCCTTGCAGATATTAA GAAGTTTGCTAGCGCTGTTTCTGTCAACACCCAATCCGTTTTCCCAACAACTAACCAAT TCTTGATAAACCAGACCAACAAGCTTGTTCCCACCCTGCAATCTGCTGGCCTTTCAGTT TATGCTTATGTGCTCATGAATGAGTTCACTTCTCAACCATATGACTTCTTCTCGGACGCC ACCGCACAGATCAATGCGTATGTGCAAAGTGCTAAGGTGGACGGAATCATCACTGATT TCCCTGGGACTGCTCACAGATACAAATTGAACTCCTGCATGGGAAAGAATGCACCTGA CTTTATGCGGCCTGCGCAGCCAGGGGGGCTCATTTCAGTCATGGACCAACGTGCTCAG

CCGCCAGCGGCGGCTCCAATGCCACTCTTGACAGACTCTGACGTCGCAGAACCACCTC TACCTCCGCACTTCATGGTGGTCCTCCGGCCCGACCCGGCACGGACCACAGGCGCTGG TCTTCGATTTCCAGCCGCGGGACCCGGAGGACGTCGGCGCCGCGCTGGCGGTGCTGTC GCGGAGCGAGATACCCGGACCTCGACGTCGCGGCTGATTCGTTGCGTTTGTCTCCGAG TGGGTGTTTCAGGCGTGGTTCGGAGAAGAACGCTGCGGAGGGTCCCGGACCGGCGGT GCTGGCTCGTCGGGCACTGCTGCGACGGGGACGCCGTGGGCGCCGCCGACAGGTTCA GCGAGCGGTGGCCGACCGGCCTGGTCGTCGGGGAGCACGACTGCCGGGACTACACCA ATGGGCTGGTCGAGGTCCTGACAGGTGAAAAACGTGTCCTGGAGGCGCTCAGATCCG GCGGCAGCACCAGCATCAGCGGGGCGGCGCCGCCGTGGTATGGATGA

- SiGDPD10 SETIT_016178mg

ATGGAAAAGACATACAAGGTGCACGGAGAAGATGTCCATGGCTGGTTCTCTCTAGATT TCACCGCGGACCAGCTGATTCAGAACGTCACGTTAATCCAGAACATCTTTTCTCGCCCA AGCACATTTGATGGCTCCATGGGAATGTACACGCTTGATGATATTGTTGAACTCCGCCC CCCTCAAATATGGCTTAATGTACAGTACAATTCATTCTTTCTGGAGCACAAACTAAGTAC TGAAGATTATATATTAGGACTACCAAAAAAATTTAGCCTCACTTACATCTCCTCGACAGA GATTGATTTCTTGAAAAGCCTGGGTGGGAAACTCAAGAAAAGCAAGACGAAACTCGT CTTCCGGTTCCTCAATGAAGATGTCATTGAGCCTTCAACCAAGAAGACATATGGGGAG CTCCTGAAAGACCTGAAATCCATCAAGGATTTTGCAGTCGGGATTCTTGTCCCCAAGA CCTACATCTGGCCACTGAACAAAGATCAGTACCTGTCACCATCCACAAGTTTGGTCAA AGATGCGCATGCCCTGGGGCTCGAAGTCTATGCGTCTGGATTTGCCAATGATATCGCTA CAAGTTACAACTACAGCTATGATCCCAGTGCAGAGTACTTGCAGTTCATAGACAATTCA GACTTCTCTGTCGACGGTGTGCTCACGGACTTCCCGCCCACTGCATCAGGAGCTATTGC TTGCTTGGCTCATTCTAAGGGCAATCCTCTAGCACCTCCTGGAAAGGACACCAGGCCG CTGATCATCACCCACAATGGGGCTAGCGGTGTCTTTGCTGGGAGCACAGATCTTGCCTA CCAGGAGGCGATCAAAGATGCCGCCGACATCATAGATTGCTCGGTTCAGATGTCAAAA GACGGAGTGGCCTTCTGCATGCACTCTGCCGATCTCTCCCCCCACACGACCGCGGCCA CCGCTTTCGTGTCCAAAAGCTCCACTGTCCATGAGATTCAGAACAAGTCTGGCATCTTC TCGTTCGAACTGTCATGGAGTGAGATCCAAACATTGAAGCCCGATATTTTCTCTCCGTT TGCTCAGGCAGGCCTGAAGAGAAATCCTGCATCGAAGAATGCCGGCAGGTTCCTGAC CTTGCCCCAATTCCTAGACATGGCCAAGGCCAGCAACGTCTCCGGCATACTGATCGAA ATGGAGCATGCCTCATATCTCGCCAAGAGAGGTCTCGGTGTGGTGGAGTCGGTGTCCA GCGCGCTAACCAAGGCCGGCTACGACAAGGAGACCAAGCAACAGGTGTTCATCCAGT CCGATGACTCGTCGGTGCTCTCGGCGTTCAAGAAGTTCCCGGCGTTCAAGCGGGTGCT CAACCTCGAGATGGAGTTTAGCGGCGCCTCCCAGCCGTCGCTGGACGACATCAAGAAA TTCGCCGACGGGGTGAGGATCCACCGGAGCTCGGTGGCGCAGATCACCGGGTACTTTA TGACGCGGTTCACCGACACGGTCGGCAGCCTGCAGGCCGCCAACCTCACCGTGTTCAT CGGCGTGCTCAAGAACGAGTTCATGAACCTTGGCTTCGACTACTTCGCTGACCCGACC GTCGAGATCGTCACCTACTCCTCGGCGGTGATGGCAGACGGGCTCATCACCGACTACC CTGCCACTGCAGCTTCATACTTCAGGAGTCCATGCAGTGACATGAGCCTGAACCTGAG TTACTCGATCCTGCCAGCGCAACCCGGTGCTCTGGTCCACCTGGCAGCCCCCGGGGCG CTAGCACCGGCGGCAGGGCCGGCGCCGTTGCTGGAACCCAAGGACGTCGTGGACCCG CCGCTGCCTTCTGTCAAAGCTGTGATCGCCGCCGACGCGCTGGCGCCGACGGGGACC

GCTGACAACACCTCCTCGGCGGCCAGCTACAACGCCGGGAAGAACAGCCTCTTGGGA GCTGGCATCATTGCCCTCTTGTCCCTGAGCTTCCTGCACTGA

>SiGDPD11 SETIT_006074mg

ATGGGAGTAAGATATCCTTGTATGTTTCTTGTCCTGCTATTGCTTCATGGAGCTAATGCT GCTTTGAAGGACCCAGTACAGAAATGGCAGACTCTAAGCGGTGCTCCTCCATTAGTCA TAGCTCGTGGCGGGTACTCTGGGTTGTTCCCTGATTCAAGCCACATTGGGTACCAGTTT GCCCTGGAGAATAGTCTCCCTGAAGCTGTTCTTTATTGCGACCTGCTACTTTCTAGCGA CAACGTTGGATTCTGCCATAGTTGCTTGGCACTTGATAACTCAACGCTAATAGCTGAGG TCTTCCCTAAGAATGGGAAGACATACAAAGTGAACGGAGAAGATCGGCATGGATGGTT TTCAATTGACTTCACTTCAAATCAGCTAATGCACAATGTCACATTGATCCAGAACGTTC TGTCTCGCCCGAGCATATTTGATGGCACCATGGGAATGAGCCTTGTTGATGATGTAGTA GGACTCCACCCCCCTCAACTTTGGATTAATGTACAGTATGGCCAGTTCTTCCAAGATCA CAAATTAAATATCGGAGAGTATATATTATCTAAAGTGAAAGAATTTGGGTTCAATTATGT CTCCTCACCTGAAGTTGGATTCTTGAAAACCCTTGGTGGGAAGCTCGGGAAAAGCAAT GTGAAGCTCGTTTTACAGTTTCTTGACGAGCAAGTCACTGAACCTTCCACAAAACAAA CCTATGGAGCCATTCTAAAGGACTTGAAATCTATCAAGACTTTTGCCTCAGGAATTCTC GTCCCCAAGACCTACATTTGGCCTGTGAACAAGGATCATTACTTGCAGCCAGCTACTAA TTTGGTGAAAGATGCTCATGCCCTAGGCTTGGAGGTCTATGCGTTTAAGTTTGCCAATG ATGACATCTCAAGTTACAACTACAGCTACGATCCTAGTGCCGAGTACTTGCAGTTTATT GATAATTCTGACTTCTCTGTTGATGGTGTCCTCACGGACTTCCCGTCCACAGCTTCAGC AGCCGTAGCCTGTTTAGCACATACTAAGCACAACCCGCTTCCTCCTCCCGGAAATGATA CCAGGCCACTGATCATCACCCACAATGGTGCAAGTGGCATCTTCCCTGGTGGCACTGA CCTTGCGTATCAACAAGCAGTGGAAGACGGCGCAGACATAATCGACTGTTCAGTACAG ATGTCAAAGGACGCAGTGCTATTCTGCCTGGACTCTCCAGATCTTACAAAAGGCACGA CAGCGGCAACAATGTTCACGACAAAAGTTGCCACTGTGAATGAAATACAGAACGGGT CTGGCATCTTCTCATTTGATCTTTCATGGAGCGAGATCCAAACTCTAAAGCCTGATCTC GTTGGCCCGTTCAGTCAAGCCGGACTGAAAAGAAATCCAGCAGCAAAGAACAGCGGG AAACTCATGACATTAGCTGAGTTCCTGGCCTTCTCAAAGAGCAGCAACGTCTCTGGCA TACTAGTTGACATTCGTAACGCTCCATACCTTGCAACCAGAGGCATTGGCATTGTCGAT GCCATCTCCAGCGCACTCGTCAATGCCAGCTACGACAAAGAGACGAGGCAGCAGGTG CTCATCGCGTCGGACGACACCGCCGTGCTCGGATCATTCAACAACTTCCCGGCGTTCA AGCGGGTCCTCCAGATCGGCAACGTGATCAGCGACGTGTCCAGGGCGTCCGTGGAGG AAGTGGCCAAGTTTGCGGATGCCGTGTCGATCACCCGTGGCTCGGTTGTCCAGGCGCA AGGCTCATTCCTCGTGCGGTTCACCGACGTGATCGACAAGATGCATGCCGCAAACCTG TCGGTGTACGTCGGCCTGCTCAAGGACGAGTTCATGAACCTTGGGTTCGACTTCTGGG CTAACCCGATGGTGGAGATTGTGACCTACTCGTCGCTGATGGCTGATGGGATTGTGACC GAGTTCCCTGCAACGGCAGCCGAGTACTTCAGGAGCCCATGCAGTGACTTCAGTAAGA ACCTGACCTACACAATCATGCCTGCAAAACCGGGGACTTTGATCAACCTGACGGACCA CAGTGCACTGCCACCGGCGCAGGGGCCGGCGCCGGTGCTGGAACCTGCCGACGTCGT GGATCCTCCGCTGCCGGCGGTAACCGTCGGTGGTCATGGAGCTGCATCATCGTCTTCAA ATGATTCCAGCACTACGAGCAGCGCCATGGCGTCCGGCGCCAGCTCTGGCCTTTGCTT GTTGGTGGCTGGACTCGCCGTGCTTTTGGCAGTGTGCTCTCGGTGA

- SiGDPD12 SETIT_013291mg

ATGAGGAGGGGTAGAGGACATGGAGGAGGAGGGGTATCCGCGTCCCTCGCCGCGCTG CTGTGCTGCGGCTGCGTCATGGTCCTCGCCGGCGCAGCCGCCGCGCAGGGGCCCCGG CTTCCCTCCGCGTACAAAACCCTAAGAGGTGATGCTCCACGAGTTGTGGCCAAAGGCG GGTTCTCAGGAGTGTTTCCCGACTCCAGCTCAGATGCCTACTCCTTTGCGTTGATCGCC AGCGCACCCGGTACAACTCTGTGGTGTGACGTCCAGCTGACAAAGGATGGCGTTGGA GTCTGCCTTCGGGACATAAACATGAATAATTGCACCAACGTCGCCCACACTTACCAGGC GAGAAAGCGGACCTATGTCATCGATGGCGTGCGGAAAAATGGATGGTTTGCTCTGGAC TTTACCAAGGATGAGCTTCAGTCTGTTTCTTTAACACAAGCAATTTGGTCTCGCACCTA CAGATTCGATTCTGTCGGATATTCTATCCTCTCTGTCACAGACTTGCTGTCCATTGTCAA GCAGCCTTCTGTTTGGTTGAATGTTCAGCATGACACCTTCTACAAAGAACATGGTTTGA ACATGAGGAACTACATACTTTCCATCCAAAAGCGTGTATCTGTGGATTATATCTCGTCAC CTGAACTTGGCTTCCTCCAAAATATATCTGGAACAGTTCATCGCAAAACAAAGCTTGTG TTTAGCTTTCTTGATAAATCCCTCTTGGATCATTCTATAAACCAAACATATGGTTCACTTT TGAGTAACCTAACGCTTATTAAGTCTATTGCATCTGGCATAATGGTCCCGAAGACATACA TTTGGCCAGTGACAAAGGATAACTATCTACAGCCGTCCACATCAATTGTCGCCGAAGCC CACAATGCAGGGCTGGAAATATATGCCTCTGATTTTGCAAATGATAGAATTATTCCCTAT AACTACAGTTATGATCCATTGGCAGAATACCTAAACTTTATCAGCGATGGTGGCTTCTCT GTTGATGGTGTATTGTCAGAACACCCTATTACTGCATCAGAGGCTATTGGTTGCTTCGCT AATCTGAATTCAAGTGAGACTGGTCATGGGGAACCCTTAGTTATCTCGCATAATGGTGC TAGTGGAGATTATCCAGACTGTACGGACCTGGCCTACAATAGTGCAATTAATGATGGCG CAGATGTCATTGATTGTCCTGTTCAAGTGACAAGTGATGGAGTTCTTATGTGCATGAGT TCCATTAACCTGCTTGATACCACGAATGTGCAGGGAACAACTTTCAGCTCTCTTAGTTC TGTTGTTCCAGAAATTCAGGCTACAGCAGGAATCTTCACATTCAACCTTACTTGGGATG ACATTAATAGTAGCACCTTAAAACCCAAAATATCTTCCCCAGTGAGTGATTATTATCTTG TAAGGAACCCAAGGTACACAAATCAAGGGAAGTTTCTGAACTTATCTGACTTTCTAGC AATCGGAATGGATAAGGATTTGTCTGGTGTCATGATCATCATTGAGAATGCTGCATTTGT GGCAAAGTCATTAGGAATTGACATAGTTGATTCTGTAAATGCTGCCTTAAGTGCTGCTG GCTATGATAATCAGACTACCAAGCAAGTTCTGATCCAGTCGAAAGATAGTGCTGTTCTT GTCAAACTGAAGCAGCAGAAAACAAAATGCAAGCTTGTGTACACTCTCCCCTTAGGCA TTGGGGATGCTTCTACTTCCTCATTAGAAGCCATGAAGAAGTTTGCTGATGCTGTAGTT GTTGATAGGAAATCTGTTTTTACCTTAAGTCAAGATTTTGCGATCCGACAGAACAGTCT TGTGAAAGATCTACAGTCAGCAGGGCTAGCTGTGTACGCACAGGTGTTCCGGAATGAG TTTGTATCACAACCGTTGGACTTCTTTGCAGATGAAACTGTTGAGATCAATTACTATGTT CAATCATTTAATCTGTCAGGCATCATAACTGACTTCCCGAAGACAGTCAGAAGATACAA AAAAAACACTTGTACAGGTTTGGGAAATGACATGCCCAACTACATGCAGCGCATTGAC GTTGGCTCCCTTTACCAGTTACTTCAACCCTTCAAAGCCCAGCCGCCATCTGTGCCGCC AATGCCGACACTGAATGCTTCAAGCGTGGAGGAGGCACCGCTTCCTCCTGTTGCGTCA AGAAATGGGTCCGGTGGTTCCTCAAGTGGCGCCGAAACTCCTGGTGCGCCTCCTGCTG CTGCTCACAAGGCTACTGTAAGTACTGGCATGCTGTTTGTAATGGTTTTCACAGCTCTT CTGATCTGA

- SiGDPD13 SETIT_009453mg

ATGAGGGCCTGCCATGTCTGTTCCGTTCTCGCGCAGCTCATGCTGCTGTGGCTGGGCGT CGCCGCCGCGCAGAAGGCCACTTCTTGGAAGACATTGAGCGGCAAGGCTCCAGCAAT

CGTAGCCAAGGGTGGATTTTCAGGGCTGTTCCCTGATTCAAGCCCAGATGCTTATGGAT TTGTGCAGTATTCTAGCTCGCCGGATACAGTGCTATACTGTGATGTTCGGCTAACCAAG GATGAGGTTGGCTTGTGCCTGCCGGACATAAAGATGGACAACTGCACAAATATTGCAG ATATCTATGCACAGGGTCAGAAGAGCTACCTTGTCAATGGCGTGCCTACATCAGGATGG TTCTCTGTAGACTACAATAACACTGAGCTTGGACAAGTGTCTCTCATCCAGTCAATCGC TTCCCGGTCACCCAGATTTGATTCAAATTTCTATCCACCACTTGCTGTCGAAGATGTGC GGTCCAAATTCAAACCTCCTGGAATTTGGCTCAATGTTCAGCATGACAGGTTTTACAGC CAGTTCAATCTAAGCATGAGGAATTATATCATTTCTGTATCTAAGCGTGTTGTTGTCAATT ATATCTCATCACCTGAAGTGAGCTTCCTCACCAGCGTACTTGGAAGAGTTAGCAAAAA GACAAAGCTTGTGTTCCGCTATCTTGATGAGAGCACTCTCGAACCATCTATGAACCAGA CGTATGGTTCCATGTTGAAGAATCTTACATTTGTCAAGACATTTGCATCCGGGATACTTG TCCCAAAGAGCTATATTTGGCCTACTTCGGCAGATAATTATCTGCAGCCGCACACTTCA GTTGTCAATGATGCTCATAAAGCTGGGTTGGAAATTTATGCCGCTGATTTTGCAAATGA CTTTATGATCAGTTACAACTACAGCTACGATCCCTTAGCAGAATGTCTGACGTTCATTGA TAATGGTGTGTTCTCTGTTGATGGTGTACTGACTGATTTCCCTGTTACACCATCAGAGGC AATTGGGTGTTTTACCAATTTAAACAAAAGCAACACAGATCATGGAAAACCTCTTATTA TTTCCCACAATGGTGCTAGTGGGGACTACCCCGGTTGCACTGATCTAGCTTATCAAAAA GCAGTTGATGATGGCGCAGATGTCATTGATTGCCCTGTCCAAGTGACCAAAGATGGAG TGCTAGTATGCATGAGTTCAGTTAATCTAATGGATGATACTACTGTTGCAAGATCGCAAT TTGCCTCTCAGACGGCAGTGATCAAAGAAATTCAGAGTGCGCGAGGAGTCTTTACGTT CAACCTCACTTGGGATGACATTGTAAAGAATCTGAGACCCATAATATCTACCCCATTATC CACCTACAGAATGGATAGAAATCCGAGGTACAGGAATGCAGGAAAGTTCATGAGACTA TCAGACTTTTTGGACTTCACAAAGAATAAGGATTTATCAGGAATCATGATCAGTATAGA GCATGCCACTTTTGTGGCAGAGGAACTTGGATTTGACATGGTTGATACAGTTATCAAAG CCCTTGGCGATGCTGGTTACAACAATCAAACTACCCAGAAAGTTATGATTCAGTCGACG AATAGTTCAGTTCTAGAGAAGTTCAAGCAGCAAACAAAGTACGATCTTGTGTACATGA TCAACGAAGAAGTCAGGGATGCCACACCTTCTTCCCTCGTGGACATCAAAAAGTTTGC TAGTGCCGTATCCGTTGATACCAGCTCTGTTTTCCCTGAACCCCACCATTTCACAATGTA CAAGACCAATCTTGTCCAGACACTGCAGACTGCGGGTCTCTCGGTCTATGTTTACACTC TCATGAATGAGTTTGTGTCTCAGCCATACGACTTCTTCGCAGATGCGACAGTGCAGATC AATGCGTATGTTAAGGGCGCGGGAGTGGACGGGCTGATCACTGATTTTCCTGCCACAG CTCGAAGATACAAGGTGAACAGTTGCATGAACATGGGCAACAGCGCGCCGATTTTCAT GGCCCCTCCTCGTGCTGGCGATCTCATGCAAATCATCAGCAAACTTGCGCAACCACCA GCATTGGCTCCCATGCCGCTCCTCACAGACTCGGACGTGGCGGAGCCGCCCCTGCCTC CCGCCAGATCAAACAGCAGCACAGCTCCAACACATTCCGGCGCAACCAGGATGCATG CTCATGCTACGCACATCCCGGTCCTTGTCACACTGGCGGTGCTTTTCGCTTGGTGTTCC CTGGTCTGA

- SiGDPD14 SETIT_025849mg

ATGAGGAGGGGTAGAGGACATGGAGGAGGAGGGGTGTCCGCGTCCCTCGCCGCGCTG CTGTGCTGCGGCTGCGTCATGGTCCTCGCCGGTGCAGCCGCCGCGCAGGGCCCCTGGC TTCCCTACGCGTACAAAACCCTAAGTGGTGATGCTCCACGAGTTGTGGCCAAAGGCGG GTTCTCAGGAGTGTTTCCCGACTCCAGCTCAGATGCCTACTCCTTTACGTTGATGGCCA GCGCAGCCGGTACAACTCTGTGGTGTGACGTCCAGCTGACAAAGGATGGCGTGGGAG

TCTGCCTTCGGGACATTAACATGGATAATTGCACCAACGTCGCCCAAGCTTACCCTGCG AGAAAGCGGACCTATGTCATCGATGGCGTGCGGAAAAATGGATGGTTTGCTCTGGACT TTACCAAGGATGAGCTTCAGTCTGTTATTTTAACACAAGCAATTTGGTCTCGCACCTAC AGTTTCGATTCTGTCGGGTATTCTATCCTCTCTGTCACAGACTTGCTGTCCATTGTCAAG CAGCCTTCTGTTTGGTTGAATGTTCAGCATGACACCTTCTACAAAGAACATGGTTTGAA CATGAGGAACTACATATTTTCCATCCAAAAGCGTGTATCTGTGGATTATATCTCGTCACC TGAACTTGGCTTCCTCCAAAATATATCTGGAACAGTTCGTGGCAAGACAAAGCTTGTG TTTAGCTTTCTTGATAAATCCCTCTTGGATCATTCTATAAACCGAACATATGGTTCACTTT TGAGTAACCTAACGCTTATTAAGTCTATTGCATTTGGCATAATGGTCCCGAAGACATACA TTTGGCCAGTGACAAAGGATAACTATCTACAGCCGTCCACATCAATTGTCGCCGAAGCC CACAATGCAGGGCTGGAAATATATGCCTCTGATTTTGCAAATGATAGAATTATTCCCTAT AACTACAGTTATGATCCATTGGCAGAATACCTAAACTTTATCAGCGATGGTGGCTTCTCT GTTGATGGTGTATTGTCAGAACACCCTTTTACTGCATCAGAGGCTATTGGTTGCTTGGCT AGTCTGAATTCAAGTAAGACTGATCATGGGGAACCTTTAGTTATCTCGCATAATGGTGC TAGTGGAGATTATCCAGACTGTACGGACCTGGCCTACCATAGTGCAATTAATGATGGCG CAGATGTCATTGATTGTCCTGTTCAAGTGACAAGTGATGGAGTTCTTATGTGCATGAGT TCCATTAACCTGCTTGATACCACGAATGTGCAGGGAACAACTTTCAGCTCTCGTAGTTC TGTTGTTCCAGAAATTCAGGCTACAGCAGGAATCTTCACATTCAACCTTACTTGGGATG ACATTAATAGTAGCACCTTAAAACCCAAAATATCTTCCCCAGTGAGTGATTATTATCTTG TAAGGAACCCAAGGTACACAAATCAAGGGAAGTTTCTGAAGTTATCTGACTTTCTAGC AATCGGAATGGATACAGATTTGTCTGGTGTCATGATCATCATTGAGAATGCTGCATTTGT GGCAAAGTCATTAGGAATTGACATAGTTGATTCTGTAAATGCTGCCTTAAGTGCTGCTG GTTACGATAATCAGACTACCAAGCAAGTTCTGATCCAGTCGAAAGATAGTGCTGTTCTT GTCAAACTGAAGCAGCAGAAAACAAAATGCAAGCTTGTTTACACTCTCCCCTTAGGCA TTGGGGATGCTTCTACTTCCTCATTAGAAGCCATGAAGAACTTTGCTAATGCTGTAGTT GTTGATAGGAAATCTGTTTTTACCTTAAGTCGAGCTTTTGCGATCCGACAGAACAGTCT TGTGAAACATCTACAGTCAGCAGGGCTAGCTGTGTACGCACAGGTGTTCCGGAATGAG TTTGTATCCCAACCGTTGGACTTCTTTGCAGATGAAACTGTTGAGATCAATTACTATGTT CAATCATTTAATCTGTCAGGCATCATAACTGACTTCCCGAAGACAGTCAGAAGATACAA AAAAATGCCCGACTACATGCAGAGCATTGACGTTGGCTCCCTTTACCAGTTACTTCAAC CCTTCAAAGCCCAGCCGCCATCTTTGCCGCCAATGCCGACACTGAATGCTTCAAGCGT GGAGGAGCCACCGCTTCCTCCTGTTGCGTCAAGGAAGAGAGATCTTTTACGGTACACA AACACAAGACTACCTTCTCTTGCTATGGGGGTTGGCATTGGTATGTTCGTTTTGCTTCTG GTTCTTGCTGCCATCTTTGCAACAAAAAGACTCAAGATTCGTAAGGCCAGAAAAATGA GGGAGAAATTCTTCAAGCGAAACCGCGGGTTGCTGCTTCGACAACTAGTAGACAAGG ACATTGCCGAAAGGATGATCTTCAGCTTGGAAGAGCTCGAAAAGGCAACAAATAAGTT TGATGAGGCTCGCATCCTGGGCGGCGGAGGGCACGGCACGGTGTACAAAGGCATACT GTCAAACCAGCGTGTTGTTGCCATTAAAGTGTCAAGGCTTGTAGTTCAAAGAGAGATC GATGAATTCATAAACGAAGTTGCCATCCTTTCGCAAATAAACCATAGAAATGTAGTGAA GCTTTTTGGGTGTTGCCTTGAGACAGAAGTTCCATTGTTGGTCTACGAGTTCGTTCCAA ATGGCACACTTTACGACCACCTTCATGTTGACAACCCTCAAAGACCACTGACATGGAA AGATCGGCTGCGTATCGCCTCTGAAGTTGCCAGTTCTCTCGCCTATCTTCACTCAGCTG CTTCGACATCGGTTGTGCACAGGGACATCAAGACATCCAACATACTACTTGATGATCGA TTGACAGCCAAGGTTTCGGACTTCGGTGCCTCGAGAGGCATCGCAATCGATCAATCGG

GAGTGACAACCGGCATACAAGGAACATTCGGCTATTTGGATCCCGAGTACTACTACAC AAGGCGATTGACCGATAAAAGTGATGTCTACAGCTACGGCGTCATGCTTGTTGAACTTC TAACAAGAACGAAAATTATGTACATTTCGCCTGAAGGTGTGAGCCTCGTGGCACACTT TGTCACGTCCCTGGATCAAGGTGAGCTCAATGAGATACTAGATGAGCAGGTCATCGAA GAAGGAGAAGAGGAGGGCAAACAAGTGGCAGAAATAGCAGCAATGTGCTTGAGGAT GAAGGGAGAAGACAGGCCAACCATGCGGAGCGTGGAGATGAGGCTTCAAGGGCTGC TGGGTTCAGAGATTAACACTTCAGTGATTGGAGAGGGTCATGTGAATGGACTCAACGG CCTAACATTTCAAGGAGGAAATGCCAATGCCGGCGACAACTACTGCAGTAGACGGTAC AGCATCGAGGAAGAGATCTTAGTTTCTGCGAGCTTGGAGCGCTGA
