# Supplementary material for: Genome-wide identification of GDPD gene family in foxtail millet (Setaria italica L.) and functional characterization of SiGDPD14 under low phosphorus stress
Source: Front Plant Sci. 2025 Jun 18;16:1586547. doi: 10.3389/fpls.2025.1586547 (PMC12213840; doi:10.3389/fpls.2025.1586547)
Supplement: Supplementary file 3 [file DataSheet3.docx]

The protein sequences of GDPD family in Setaria italica

>SiGDPD1 SETIT_017495mg

MAQLKAARVADVPNLDVVAPGLVVAAGESDAAAIAAAGKRGPAGGGGGRFSVIGHRG KGMNALASADRRLQEVRENTVRSFNDAARFPVDYVEFDVQVTKDGCPIIFHDNFIYTEED GKISQKRVTDLQLEDFVQYGPQNEKGKIGKPLLRKMKDGRIINWNVQSEDALCTLQEAFE KVNTRLGFNVELKFDDNLEYQEEELTRILQAILKVIFEHAKDRPILFSSFQPDAALLMRKL QSKYPVSMLNTAQAFVFMFMNSSTALIISFMLFNQVYFLTNGGTEIYIDVRRNSLEEAIKL CLSSGLQGIVSEARGIFRHPAAIPKIKEANLSLLTYGTLK

>SiGDPD2 SETIT_020031mg

MGMYTLDDIVELRPPQIWLNVQDTRPLIITHNGAGGVFAGSTDLAYQEAIKDAADIIDCS VQMSKDGVAFCMHSADLPPPQTHEKNRSSILVRDARVQHASYLAKRGLGVVESVSSALT KAGYDKETKQQVFIQSDDSSVLSAFKKFPAFKRVLNLEMEFSGASQPSLDDIKKFADGVR IHRSSVAQITGYFMTRFTDTVGSLQAANLTVFIGVLKNEFMNLGFDYFADPTVEIVTYSSA VMADGLITDYPATALQLHTSVRSPCSDMSLNLSYSILPAQPGALVHLAAPGALAPAAGPA PLLEPKDVVDPPLPPVKAVIAADAPAPKGTADNTSSAASFNAGKNSLLGAGIVALLSLSF LH

>SiGDPD3 SETIT_032348mg

VVAARPLVGEGGGGAWGDSRAPLQTSRPFNIAHRGSNGEFPEETAAAYARAIDEGADFIE ADIEATRDGHLVCFHDTTLDDATDVADHPEFAGRRRTLEVQWANVTGYFITDFTLAELK TLRAKQRWDFRDKSHDGISPIITFEEFIDIALNAKRVVGIYPEMKNPAFMNKHVRWADGK RYEDKFVATLKKYGYGGRYMSPAWRAKPVFVQSFAPTSLVRAAGLTDSPLIFLIDDVTV RTEDTNQSYDEITSGEYLDYMSKYVVGIGPWKDTVVPPTKDNRLATPTDLVAMAHARGL QVHPYTYRNENKFLHFNFRQDPYAEYDYWINDVGVDGLFTDFPASLRRFQEWTAKKKD

>SiGDPD4 SETIT_000875mg

MALSRSSRMVAGHLSRYSSAFGSALRYSGEAQGPVISHGFLRNLRRPWNSDLPAPAAEEA AEAIEVGAVVGSTELGGESGNGGDRPMRREHAGNRDGLGCCWAENHGAWSRAVSSSAP LKLRSSLPCSLAYSGESPGGTGGKWSKKSVGKRGEGALLQTCTMRPSRCCVPLFLLLLSH LAFARPLFPLPSKTKNEEKRPIQTFRPYNIAHRGSNGEIPEETAVAYLRAIEEGADFIETDI LASKDGTLICFHDVTLDETTDVAEHKEFANRRRTYEVEWSNVTGWFVVDFTLEELKTLK VKQRYSFRDQQYNGMFSIITFEEFISIALDADRTVGIYPEIKDPVFINKHVKWADGKKFED KFVDTLLKYGYKGQYMSENWLKQPLFIQSFAPTSIIYVSKLIDSPKVFLIDDITVRTQDTNQ SYWEITSDDYLAYIGKYVVGLGPWKDTIVPAAGNYLMPPSDLVARAHAHNLQVHPYTY RNENQFLHFNFHQDPYAEYDFWIKNVGVDGLFTDFTGTLHQYQELTSPHRKDETANSLL VKISQMISAYEGL

>SiGDPD5 SETIT_014004mg

MALLPAATRAAAAGLCRIAASASSSPPSSAPARRERELPLALLAERGKMVVGGHRGMGM NAVGAPPGARVGAARERENTLLSFGRAAAHAAVAFVEFDVQVTKDGCPIIFHDDFILTQG IGAVYERRVTDLLLEEFLSYGPQKESRKVSKPLLRRTGDGRVLNWSTEEDDSLCTLQEVF

ECVSPHLGFNIELKFDDNVLHHKKDLERALQAILQVVFQNARDRPVFFSSFHPDAAMMTR ELQSLYPVLFLTEGGTSKHHDSRRNSLNNAIQVCLEHDLHGIVSDVRGILKNPAAVVRAE SNLALLTYGQLK

>SiGDPD6 SETIT_010283mg

MAQRKAARAAADAAVPPAMPEPDIAAELHALVASSTFPAPCIGGAAAARDDAGRRRPPL VVIGHRGKGMNALASPDPRVRGDVRENTLRSLNAAAASHPTVAYVEFDVQVTKDGCPVI FHDNFIYTQENGEISGRRVTELHLDEFLSYGPQRNQEKAGKPLLRKLKDGRILKWDVQKE DALCTLREAFEGVDRRVGFNVELKFDDDLAYTEEALTGVLQAVVFEHADGRPVIFSSFKP DAALLIRKLQDKYPVYFLTNGGTQIYADPRRNSLEEAVRLCVAGSLQGIVSEVRAILRQPA AAAKIKEAGLSLMTYGQLNNVPEVVFVQHLLGVDGVIVDLVREIAEAVSAFSAAAREPSP ESCGEVERLETAAAGTPSFSPREMSFLLRLIPELVQ

>SiGDPD7 SETIT_0280281mg

MEAFRMALDARVDCMEVDVSRSSDGVLFALHDSCSNPGGSPIKHTVIAMSPDGPGSKDD KRVKAFAHITIHIILRSRPPVIQRYISWLRSKGRETCILQDGLLICRDLQKMSGNSTAKVG HWSTDEIKALRFQLSKRVQNEEVPKAEDALAVISRSVRQVILDVKVGPPSFDKGLAEDVL SLLKRTNCKNCLVWAKTDNLARDIIKLSEDVVVGYIVMVDKSTGRKTEFVRLEGAKVAG VYHPLIHEKVVKVMRRHGKKVFAWTVDDNKSMEKMLHEHVDAIVTSNPSLLQQLMHE TRTECLEDGFALP

>SiGDPD8 SETIT_036072mg

MASSWYTSIIVLVLLFGGSKANPAATSHGQLDVNHKKPLQTFRPHNIAHRGSNGELPEET AAAYLRAIEEGADFIETDILASKDGHLICFHDVTLDATTDVANRREFANRKRTYEVEGAK MTGWFVVDFTLKELKSLRVKQRYSFRDQRYNGKYQIITFEEYILIALYADRIVGIYPEIKNP VFINQHVKWSNGKNFEDKFVEILLKYGYKGEYMSEDWFKQPLFIQSFAPTSLIYISNMTNS PKVFLIDDTTVRTQDTNQSYYEITSNAYFAFIRNYVIGIGPWKDTIVPPKNNYLGQPTDLV ARAHALNLQVHPYTFRNENSYLHFNFNQDPYVEYEYWLNEIGVDGLFTDFTGSLHKYQE WTTPYPKKEKNAEALLHEIANMLKADGY

>SiGDPD9 SETIT_016242mg

MADRVAGRRRKQNQSRAHDARLLHETTCAVSHWADARGPGDRGAGRLAPPPAPRALFR SARTPRPHAHRPQATAQKKKRVPWNFRNFCYILLPKAEQHSNNTQANIVYKSVVLSSIIAS PPVSISLSLSLVLKIERGRRTRIALPPSPVRLPCSSASLPGPGVLGDTASGAAATAAADAA AGGLVGGGSAMGRGSRACSVLGSALLLLLVSLGSAAAQKGSTWKTLSGKAPVIIAKGGF SGLFPDSSDLAYQFVPIASSPDTALLCDVRLTKDGAGICLPNIKMDNCTFISDVFPQGKST YNVNGVSTTGWFSVDFTSTDLQNVTLRQSIFSRPSYFDGSMQIVPVEAVLSVFKAPAVWL NVQHDSFYSQFKLSMRSYILSLSKQYIADYISSPEVNFLTSISGRVSKRTKLVFRFLDER SIEPSTNQTYGSMLKNLTFVKTFASGILVPKNYIWPVTPDNYLLSYTSVAADAHKAGLEI YAADFANDFTISYNYSYDPLAEYLYFIDNDAFSVDGVLTDFPITPSEAVGCFSNLNNSKT DHAKPLVISHNGASGDYPDCTDQAYQKAVDDGADVIDCPVQVTKDGIPICMSSIDLIDVT NVAKSEFASQTTTINDLKAGPGVFTFNLTWDDISKNLKPMISSPMNKYLLFRNPRNKNAG NFMRLSDFLAFAKDKDLSGIMITVENAAFMAEKLGFGVVDAVIKALDDSGYNKQTAQK VMIQSTNSSVLVKFKQETKYDLVYMIEESVRDAAPSSLADIKKFASAVSVNTQSVFPTTN

QFLINQTNKLVPTLQSAGLSVYAYVLMNEFTSQPYDFFSDATAQINAYVQSAKVDGIITDF PGTAHRYKLNSCMGKNAPDFMRPAQPGGLISVMDQRAQPPAAAPMPLLTDSDVAEPPLP PVSNTTTASSPSHAALRMKTDVSILVTLLVLCASLLI

>SiGDPD10 SETIT_016178mg

MQGAPPEQMGASYPHMFLIFLLFHGANAASNAPAGPKWQTLSGRPPQVIARGGFSGLFP DSSQYAYQFALSTSLPDVVLFCDLQFSSDSTGFCKTGLTLDNSTTVSEVFPKMEKTYKVH GEDVHGWFSLDFTADQLIQNVTLIQNIFSRPSTFDGSMGMYTLDDIVELRPPQIWLNVQY NSFFLEHKLSTEDYILGLPKKFSLTYISSTEIDFLKSLGGKLKKSKTKLVFRFLNEDVIEP STKKTYGELLKDLKSIKDFAVGILVPKTYIWPLNKDQYLSPSTSLVKDAHALGLEVYASG FANDIATSYNYSYDPSAEYLQFIDNSDFSVDGVLTDFPPTASGAIEDTRPLIITHNGASG VFAGSTDLAYQEAIKDAADIIDCSVQMSKDGVAFCMHSADLSPHTTAATAFVSKSSTVHE IQNKSGIFSFELSWSEIQTLKPDIFSPFAQAGLKRNPASKNAGRFLTLPQFLDMAKASNV SGILIEMEHASYLAKRGLGVVESVSSALTKAGYDKETKQQVFIQSDDSSVLSAFKKFPAF KRVLNLEMEFSGASQPSLDDIKKFADGVRIHRSSVAQITGYFMTRFTDTVGSLQAANLTV FIGVLKNEFMNLGFDYFADPTVEIVTYSSAVMADGLITDYPATAASYFRSPCSDMSLNLS YSILPAQPGALVHLAAPGALAPAAGPAPLLEPKDVVDPPLPSVKAHPAWRHTTYTGADT HTTSSSFLPWRRSPRTPAPLRRRLLAYTAATARPPLPEPGGPPPLHAVALGGYIYSSRNLS CLVSGTAKASVSGAETSSGGEDVNEIIGAVEAVESTTPGASFLAKVAIAIGIAATVTVIS LVRKQPSSGPSFSLPQIVDASTQSDAAAATLGYSFSAFGKKVIIPEYTPGGVCLSDMIPF FLGKLFRQTKASEGISSKIGIGKDKALSISRAVQKYGNLIGFVERFSIGVRNITAFLAGA LGIPADCYFAGVCFGCLLTLPIQGHNAPWKHTMEGPDDMPAHIKSSMFGCALTIPITDGR LNMGTWQGIWLCEHRDHASPRKIVITLNGV

>SiGDPD11 SETIT_006074mg

MSHNVLSRPSIFDGTMGMSLVDDVVGLHPPQLWINVQYGQFFQDHKLNIGEYILSKVKEF GFNYVSSPEVGFLKTLGGKLGKSNVKLVLQFLDEQVTEPSTKQTYGAILKDLKSIKTFAS GILVPKTYIWPVNKDHYLQPATNLVKDAHALGLEVYAFKFANDDISSYNYSYDPSAEYL QFIDNSDFSVDGVLTDFPSTASAAVACLAHTKHNPLPPPGNDTRPLIITHNGASGIFPGGT DLAYQQAVEDGADIIDCSVQMSKDAVLFCLDSPDLTKGTTAATMFTTKVATVNEIQNGS GIFSFDLSWSEIQTLKPDLVGPFSQAGLKRNPAAKNSGKLMTLAEFLAFSKSSNVSGILVD IRNAPYLATRGIGIVDAISSALVNASYDKETRQQVLIASDDTAVLGSFNNFPAFKRVLQI GNVISDVSRASVEEVAKFADAVSITRGSVVQAQGSFLVRFTDVIDKMHAANLSVYVGLL KDEFMNLGFDFWANPMVEIVTYSSLMADGIVTEFPATAAEYFRSPCSDFSKNLTYTIMPA KPGTLINLTDHSALPPAQGPAPVLEPADVVDPPLPAVTVGGHGAASSSSNDSSTTSSAMAS GASSGLCLLVAGLAVLLAVCSR

>SiGDPD12 SETIT_013291mg

MRRGRGHGGGGVSASLAALLCCGCVMVLAGAAAAQGPRLPSAYKTLRGDAPRVVAKG GFSGVFPDSSSDAYSFALIASAPGTTLWCDVQLTKDGVGVCLRDINMNNCTNVAHTYQA RKRTYVIDGVRKNGWFALDFTKDELQSVSLTQAIWSRTYRFDSVGYSILSVTDLLSIVKQP SVWLNVQHDTFYKEHGLNMRNYILSIQKRVSVDYISSPELGFLQNISGTVHRKTKLVFSFL DKSLLDHSINQTYGSLLSNLTLIKSIASGIMVPKTYIWPVTKDNYLQPSTSIVAEAHNAGLE IYASDFANDRIIPYNYSYDPLAEYLNFISDGGFSVDGVLSEHPITASEAIGCFANLNSSETGH

GEPLVISHNGASGDYPDCTDLAYNSAINDGADVIDCPVQVTSDGVLMCMSSINLLDTTNV QGTTFSSLSSVVPEIQATAGIFTFNLTWDDINSSTLKPKISSPVSDYYLVRNPRYTNQGKFL NLSDFLAIGMDKDLSGVMIIIENAAFVAKSLGIDIVDSVNAALSAAGYDNQTTKQVLIQSK DSAVLVKLKQQKTKCKLVYTLPLGIGDASTSSLEAMKKFADAVVVDRKSVFTLSQDFAI RQNSLVKDLQSAGLAVYAQVFRNEFVSQPLDFFADETVEINYYVQSFNLSGIITDFPKTVR RYKKNTCTGLGNDMPNYMQRIDVGSLYQLLQPFKAQPPSVPPMPTLNASSVEEAPLPPV ASRNGSGGSSSGAETPGAPPAAAHKATVSTGMLFVMVFTALLI

>SiGDPD13 SETIT_009453mg

MRACHVCSVLAQLMLLWLGVAAAQKATSWKTLSGKAPAIVAKGGFSGLFPDSSPDAYG FVQYSSSPDTVLYCDVRLTKDEVGLCLPDIKMDNCTNIADIYAQGQKSYLVNGVPTSGW FSVDYNNTELGQVSLIQSIASRSPRFDSNFYPPLAVEDVRSKFKPPGIWLNVQHDRFYSQF NLSMRNYIISVSKRVVVNYISSPEVSFLTSVLGRVSKKTKLVFRYLDESTLEPSMNQTYGS MLKNLTFVKTFASGILVPKSYIWPTSADNYLQPHTSVVNDAHKAGLEIYAADFANDFMIS YNYSYDPLAECLTFIDNGVFSVDGVLTDFPVTPSEAIGCFTNLNKSNTDHGKPLIISHNGA SGDYPGCTDLAYQKAVDDGADVIDCPVQVTKDGVLVCMSSVNLMDDTTVARSQFASQT AVIKEIQSARGVFTFNLTWDDIVKNLRPIISTPLSTYRMDRNPRYRNAGKFMRLSDFLDFT KNKDLSGIMISIEHATFVAEELGFDMVDTVIKALGDAGYNNQTTQKVMIQSTNSSVLEKF KQQTKYDLVYMINEEVRDATPSSLVDIKKFASAVSVDTSSVFPEPHHFTMYKTNLVQTLQ TAGLSVYVYTLMNEFVSQPYDFFADATVQINAYVKGAGVDGLITDFPATARRYKVNSCM NMGNSAPIFMAPPRAGDLMQIISKLAQPPALAPMPLLTDSDVAEPPLPPARSNSSTAPTHS GATRMHAHATHIPVLVTLAVLFAWCSLV

>SiGDPD14 SETIT_025849mg

MRRGRGHGGGGVSASLAALLCCGCVMVLAGAAAAQGPWLPYAYKTLSGDAPRVVAK GGFSGVFPDSSSDAYSFTLMASAAGTTLWCDVQLTKDGVGVCLRDINMDNCTNVAQAY PARKRTYVIDGVRKNGWFALDFTKDELQSVISVTQAIWSRTYSFDSVGYSILSVTDLLSIV KQPSVWLNVQHDTFYKEHGLNMRNYIFSIQKRVSVDYISSPELGFLQNISGTVRGKTKLV FSFLDKSLLDHSINRTYGSLLSNLTLIKSIAFGIMVPKTYIWPVTKDNYLQPSTSIVAEAHN AGLEIYASDFANDRIIPYNYSYDPLAEYLNFISDGGFSVDGVLSEHPFTASEAIGCLASLNS SKTDHGEPLVISHNGASGDYPDCTDLAYHSAINDGADVIDCPVQVTSDGVLMCMSSINLL DTTNVQGTTFSSRSSVVPEIQATAGIFTFNLTWDDINSSTLKPKISSPVSDYYLVRNPRYT NQGKFLKLSDFLAIGMDTDLSGVMIIIENAAFVAKSLGIDIVDSVNAALSAAGYDNQTTK QVLIQSKDSAVLVKLKQQKTKCKLVYTLPLGIGDASTSSLEAMKNFANAVVVDRKSVFT LSRAFAIRQNSLVKHLQSAGLAVYAQVFRNEFVSQPLDFFADETVEINYYVQSFNLSGIIT DFPKTVRRYKKMPDYMQSIDVGSLYQLLQPFKAQPPSLPPMPTLNASSVEEPPLPPVASR KRDLLRLKIRKARKMREKFFKRNRGLLLRQLVDKDIAERMIFSLEELEKATNKFDEARIL GGGGHGTVYKGILSNQRVVAIKVSRLVVQREIDEFINEVAILSQINHRNVVKLFGCCLET EVPLLVYEFVPNGTLYDHLHVDNPQRPLTWKDRLRIASEVASSLAYLHSAASTSVVHRDI KTSNILLDDRLTAKVSDFGASRGIAIDQSGVTTGIQGTFGYLDPEYYYTRRLTDKSDVYS YGVMLVELLTRTKIMYISPEGVSLVAHFVTSLDQGELNEILDEQVIEEGEEEGKQVAEIA AMCLRMKGEDRPTMRSVEMRLQGLLGSEINTSVIGEGHVNGLNGLTFQGGNANAGDNY CSRRYSIEEEILVSASLER
