# Supplementary material for: Genome-wide identification of GDPD gene family in foxtail millet (Setaria italica L.) and functional characterization of SiGDPD14 under low phosphorus stress
Source: Front Plant Sci. 2025 Jun 18;16:1586547. doi: 10.3389/fpls.2025.1586547 (PMC12213840; doi:10.3389/fpls.2025.1586547)
Supplement: Supplementary file 4 [file DataSheet4.docx]

Supplementary Material

|  | Gene | Forwad primer (5' to 3') | Reverse primer (5' to 3') |
| --- | --- | --- | --- |
| The primers for qRT-PCR in this study | *SiGDPD1* | TGAGGAGGAACTCGTTGGAGGAG | CGTAGGTCAGCAGGGAGAGGTTAG |
|  | *SiGDPD2* | CTAACCTCTTCAGACTCCGC | CACCGTGGAGCTACCGTCTACT |
|  | *SiGDPD3* | TCACCGACTCGCCGCTCATC | GCCGACCACGTACTTGCTCATG |
|  | *SiGDPD4* | GCCCTAGATGCGGATAGAACTGTTG | ACTTCTTTCCATCAGCCCACTTGAC |
|  | *SiGDPD5* | AGTCGTCAGAGCACAAGAATCCAAC | CGCCGTTTATGCCCATCAGGTAC |
|  | *SiGDPD6* | GCCGTCCTCAAGGTTGTCTTCG | TGCGTCCCTCCGTTCGTCAG |
|  | *SiGDPD7* | CGCCTCCTCCGCCTGCTC | TGATCCACCCGCACTTCCTCTC |
|  | *SiGDPD8* | GGCTTCCTCCTGGTACACCTCTAT | TGGGCAATGTTGTGAGGTCTGAA |
|  | *SiGDPD9* | CAGTCATGGACCAACGTGCTCAG | TGCGGAGGTAGAGGTGGTTCTG |
|  | *SiGDPD10* | CGCCAACCTCACCGTGTTCATC | CCATCACCGCCGAGGAGTAGG |
|  | *SiGDPD11* | AACGGGTCTGGCATCTTCTCATTTG | CGCTGTTCTTTGCTGCTGGATTTC |
|  | *SiGDPD12* | GCTGCGGCTGCGTCATGG | CAACTCGTGGAGCATCACCTCTTAG |
|  | *SiGDPD13* | TGTGATGTTCGGCTAACCAAGGATG | GTAGGCACGCCATTGACAAGGTAG |
|  | *SiGDPD14* | GCTTCAAGGGCTGCTGGGTTC | TCCTCGATGCTGTACCGTCTACTG |
|  | *ACTIN* | GGCAAACAGGGAGAAGATGA | GAGGTTGTCGGTAAGGTCACG |
| The primers for clone *SiGDPD14* | Gene | Forwad primer (5' to 3') | Reverse primer (5' to 3') |
|  | *SiGDPD14* | CGCGGATCCATGGCTTCCTCCTGGTACACC | CTTGAGCTCTCAGTATCCATCAGCCTTCAAC |
|  |  |  |  |

# Supplementary Table1 Sequences of primers used in this study
