# Supplementary figures and images for: Genome-wide identification of GDPD gene family in foxtail millet (Setaria italica L.) and functional characterization of SiGDPD14 under low phosphorus stress
Source: Front Plant Sci. 2025 Jun 18;16:1586547. doi: 10.3389/fpls.2025.1586547 (PMC12213840; doi:10.3389/fpls.2025.1586547)

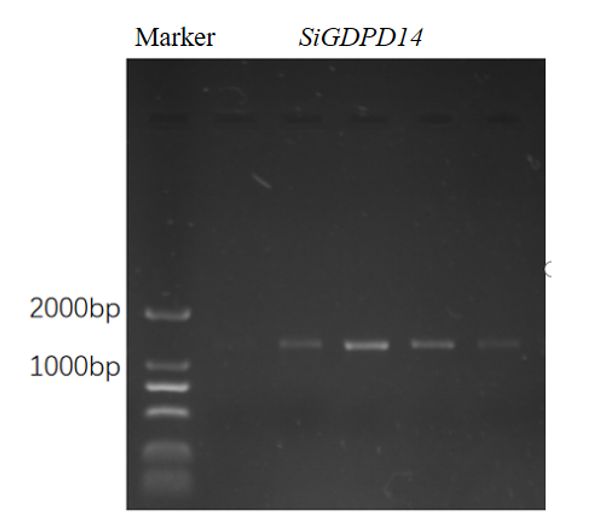

Supplement: Supplementary file 5 [file Image1.png]

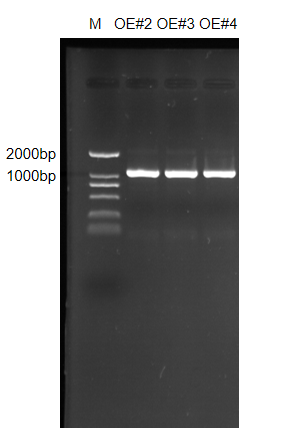

Supplement: Supplementary file 6 [file Image2.png]
